# Tau^P301L^ disengages from the proteosome core complex and neurogranin coincident with enhanced neuronal network excitability

**DOI:** 10.1038/s41419-024-06815-2

**Published:** 2024-06-18

**Authors:** Katriona L. Hole, Bangfu Zhu, Laura Huggon, Jon T. Brown, Jody M. Mason, Robert J. Williams

**Affiliations:** 1 Department of Life Sciences, University of Bath, Bath, UK; 2 Department of Clinical and Biomedical Sciences, University of Exeter, Exeter, UK; 3 Present Address: The Francis Crick Institute, London, UK; 4 Present Address: UK Dementia Research Institute at King’s College London, London, UK

**Keywords:** Molecular neuroscience, Calcium signalling

## Abstract

Tauopathies are characterised by the pathological accumulation of misfolded tau. The emerging view is that toxic tau species drive synaptic dysfunction and potentially tau propagation before measurable neurodegeneration is evident, but the underlying molecular events are not well defined. Human non-mutated 0N4R tau (tau^WT^) and P301L mutant 0N4R tau (tau^P301L^) were expressed in mouse primary cortical neurons using adeno-associated viruses to monitor early molecular changes and synaptic function before the onset of neuronal loss. In this model tau^P301L^ was differentially phosphorylated relative to tau^wt^ with a notable increase in phosphorylation at ser262. Affinity purification - mass spectrometry combined with tandem mass tagging was used to quantitatively compare the tau^WT^ and tau^P301L^ interactomes. This revealed an enrichment of tau^P301L^ with ribosomal proteins but a decreased interaction with the proteasome core complex and reduced tau^P301L^ degradation. Differences in the interaction of tau^P301L^ with members of a key synaptic calcium-calmodulin signalling pathway were also identified, most notably, increased association with CaMKII but reduced association with calcineurin and the candidate AD biomarker neurogranin. Decreased association of neurogranin to tau^P301L^ corresponded with the appearance of enhanced levels of extracellular neurogranin suggestive of potential release or leakage from synapses. Finally, analysis of neuronal network activity using micro-electrode arrays showed that overexpression of tau^P301L^ promoted basal hyperexcitability coincident with these changes in the tau interactome and implicating tau in specific early alterations in synaptic function.

## Introduction

Pathological accumulation of the microtubule-associated protein tau is closely associated with cognitive decline in Alzheimer’s disease (AD) [1, 2], and with other tauopathies, such as frontotemporal lobar degeneration-tau (FTLD-tau), which can be directly caused by mutations in the tau gene, *MAPT* [3, 4].

Tau is a regulator of axonal microtubule stability and labile domain length [5], but function extends beyond these roles and throughout different neuronal compartments. In disease, tau is enriched at the somatodendritic region and disrupts several core cellular processes, including but not limited to: microtubule stability [6], axonal transport [7], mitochondrial function [8, 9], nucleocytoplasmic transport [10], DNA protection [11], translation [12–14], and synaptic function [15]. Furthermore, in AD brain, abnormally phosphorylated, oligomeric, tau is enriched at both pre- and post-synaptic nerve terminals [16–19]. Presynaptically, the association of tau with vesicles hinders neurotransmitter release [17, 18, 20], while at dendritic spines, mutant tau impairs AMPA receptor internalisation and long-term potentiation (LTP) [21–24]. Furthermore, tau facilitates Aβ and glutamate-induced excitotoxicity as well as long-term depression (LTD) [25–28]. Tau interactome studies have been used to gain insight into the molecular mechanisms underlying these diverse effects [29–37].

Studies in AD and non-AD brain has shown differences in tau interactions at the endoplasmic reticulum [29] and differences in aggregated, phosphorylated, or soluble tau interactions [30–32]. The tau interactome has also been defined in rodent models of tauopathy including transgenic rats expressing truncated species of human tau (aa151-391) [38]; the tau^P301L^ interactome in mice exhibiting both Aβ and tau pathology [39]; and the tau^P301L/S^ interactome in rTg4510 and PS19 mouse models of primary tauopathy [33, 34]. Comparison of the wild-type and P301L mutant htau interactomes has been undertaken in undifferentiated SH-SY5Y and IMR-32 cells (human neuroblastoma-derived cell lines), differentiated ReN cells (a neural stem cell line), and human-derived iPSC neurons [35–37]. However, given the widespread use of transgenic mice for pre-clinical drug development and the absolute requirement for a model with fully functional excitatory synapses, we selected mouse primary cultured neurons as the cellular platform to directly compare the mutant tau^P301L^ and non-mutated (wild type) tau^WT^ interactomes.

Adeno-associated viruses were used to overexpress eGFP-tagged human tau^WT^ and tau^P301L^ in mouse primary cortical neurons to monitor early molecular changes occurring before the onset of neuronal damage. This demonstrated that tau^P301L^ was differentially phosphorylated relative to tau^WT^. Affinity purification – mass spectrometry (AP-MS) combined with tandem mass tagging (TMT), to quantitatively compare the wild-type and mutant tau interactomes, revealed an enrichment of tau^P301L^ with ribosomal proteins but a decrease in association with the proteasome core complex, and this corresponded with impaired tau degradation. Furthermore, differences in the interaction of tau^P301L^ with members of the calcium-calmodulin signalling pathway were identified, most notably, a loss in association with the candidate AD biomarker neurogranin matched by a parallel increase in the extracellular levels of neurogranin. Finally, analysis of neuronal network activity using micro-electrode arrays showed that overexpression of tau^P301L^ promoted basal hyperexcitability coincident with these changes in the tau interactome. Collectively, this demonstrates that the overexpression of pathological tau influences its association with specific neuronal binding partners and that this is associated with enhanced excitability in the absence of neuronal cell death.

## Materials and methods

### Culture of primary cortical neurons

Primary cortical neurons were cultured from CD1 mouse embryos at E15.5 [40] in accordance with UK Home Office Guidelines as stated in the Animals (Scientific Procedures) Act 1986, using procedures approved by the University of Bath Animal Welfare and Ethical Review Body. All tissue culture reagents were from Gibco unless stated otherwise.

Cortical tissue was collected by microdissection in PBS-Glucose (Dulbecco’s PBS, no calcium, no magnesium) supplemented with 6 mM D-(+)-Glucose (Sigma-Aldrich, G8769) removing the striatum and the meninges, and dissociated through trituration with a fire-polished pipette coated with heat-inactivated foetal bovine serum (FBS). Dissociated cells were centrifuged at 500 × *g* for 5 min and resuspended in Neurobasal medium containing B27 supplement (Serum-free) (17504044), 100 U/ml penicillin, 100 µg/ml streptomycin (penicillin–streptomycin; 15140122) and 2 mM Glutamine (25030081). Cells were seeded in Nunc™ treated multi-well plates pre-coated with 20 μg/ml Poly-D-Lysine hydrobromide (Sigma-Aldrich, P7280) and incubated at 37 °C, 5% CO_2_. For transfection, cells were seeded at ~3.5–4.5 × 10^5^ cells/ml. For AAV transduction, cells were seeded at 2 × 10^5^ cells/ml for imaging or 3 × 10^5^ cells/ml for immunoprecipitation, western blotting, and ELISA measurements. Under these growth conditions at 6–13 days in vitro (DIV) cells had a well-developed neuritic network and were 98% β-tubulin III positive and <2% positive for the astrocytic marker GFAP.

### Plasmids

pRK5-eGFP-Tau (Addgene plasmid #46904; http://n2t.net/addgene:46904; RRID: Addgene_46904) and pRK5-eGFP-Tau P301L (Addgene plasmid #46908; http://n2t.net/addgene:46908 ; RRID:Addgene_46908) were gifts from Karen Ashe [21]. pSF-SYN1 (OGS503) was purchased from Oxford Genetics. AAV-hSyn-EGFP was a gift from Bryan Roth (Addgene plasmid #50465; http://n2t.net/addgene:50465; RRID:Addgene_50465). The WPRE region was removed from all adapted constructs by restriction digest. The following constructs were generated through subcloning: pSF-hSyn1-eGFP, pSF-hSyn1-eGFP-Tau^WT^, pSF-hSyn1-eGFP-Tau^P301L^, AAV-hSyn-eGFP-Tau^WT^, AAV-hSyn1-eGFP-Tau^P301L^.

### Transduction and Transfection

pAAV-hSyn1-eGFP, pAAV-hSyn1-eGFP-Tau^WT^ and pAAV-hSyn1-eGFP-Tau^P301L^ were packaged into AAVs with the DJ serotype by Generon (Slough, UK). Viral particles were aliquoted and stored at -80°C until use where they were diluted in Neurobasal medium to the desired titre. DIV6 primary cortical neurons were transduced with AAV’s at a multiplicity of infection (MOI) of 10,000 genome copies/cell. A half media change was undertaken 2 days post infection and neurons were returned to the incubator until DIV13 -28.

Transient transfection was undertaken with Lipofectamine™2000 according to the manufacturer’s instructions with a final concentration in the well of 750 ng DNA & 2 µl Lipofectamine™ 2000/ml media.

Transfection efficiency was calculated using the Celleste Image Analysis Software (Thermo Fisher Scientific). The cell counter feature was used to count nuclei using the DAPI stain – condensed nuclei were eliminated with an upper threshold limit. GFP +ve cells were counted manually in ImageJ.

Transfection Efficiency (%) = Number of GFP+ve cells/Total Cell Number × 100

### MTT assay

Media was removed and replaced with an equal volume of pre-warmed 3-(4,5-dimethylthiazol-2-yl)-2,5-diphenyltetrazolium bromide (MTT) solution (1 mg/ml) in complete neurobasal medium. The plate was incubated for 1 h at 37 °C, 5% CO_2_. The solution was removed, and the formazan product solubilised with isopropanol and read at 595 nm using an IMark^TM^ microplate reader (BioRad). Three biological repeats were undertaken, with each experiment performed in triplicate.

### Immunofluorescence

Neurons were rinsed with PBS-Glucose and fixed with 4% paraformaldehyde (PFA) in PBS for 15 min at rtp and left in fresh PBS until use at 4 °C. PBS was replaced with blocking buffer (PBS, 5% (w/v) Bovine Serum Albumin (BSA), 0.1% Triton-X-100) for 30 min at rtp. Primary antibodies were diluted to the required concentration in antibody buffer (PBS, 1% (w/v) BSA, 0.1% Triton-X-100) and incubated at 4 °C overnight. The following antibody dilutions were used: Neurogranin (AB5620, Millipore Sigma) 1:1000; PSD95 (ab18258, Abcam) 1:1000; SV2-c (DSHB) 1:1000; Phospho-Tau Ser202, Thr205 (AT8) (MN1020, Invitrogen) 1:500; Phospho-Tau Ser262 (sc-101813, Santa Cruz Biotechnology) 1:500; GFAP (Z0334, Dako) 1:1000. Following incubation wells were washed with washing buffer (PBS, 0.1% Triton-X-100) for 10 min. Goat-derived Alexa-fluor tagged secondary antibodies (Invitrogen), diluted 1:1000 in antibody buffer, were then added and the plate was incubated at rtp in the dark for 1 h. Wells were washed with washing buffer for 10 min before addition of DAPI (Thermo Scientific), diluted 1:1000 in PBS, and further incubation in the dark for 5 min. Wells were washed once with PBS and samples were imaged using an EVOS™ M7000 imaging system.

### Western Blotting

Cells were lysed with either Radio-immunoprecipitation (RIPA) buffer (150 mM NaCl, 24 mM Tris, 0.5% (w/v) Sodium deoxycholate, 0.1% (v/v) SDS, 1% (v/v) IGEPAL in dH2O, pH 7.5) or Triton-X-100 buffer (50 mM NaCl, 50 mM Tris, 1% (v/v) Triton-X-100, pH 7.45), supplemented with 2 mM EDTA, cOmplete, EDTA-free protease inhibitor cocktail tablets (Roche) and PhosSTOP phosphatase inhibitor cocktail tablets (Roche) on ice. Cell monolayers were scraped into microfuge tubes and left for 25 min at 4 °C with constant agitation. Lysates were then centrifuged at 12,500 × *g* for 20 min at 4 °C and supernatants collected. 4× Laemmli Buffer (Bio-Rad) containing β-mercaptoethanol was added and samples were heated to 95 °C for 5 min.

Samples were resolved by 12% Tris-glycine SDS-PAGE before transfer onto 0.45 µm Amersham^TM^ Protran^TM^ nitrocellulose membrane (GE Healthcare) using the Hoefer® SemiPhor^TM^ transfer system. Following transfer, the membrane was incubated on a rocker at rtp with blocking buffer (5% (w/v) Milk Powder in Tris Buffered Saline (TBS) (50 mM Tris, 150 mM NaCl in dH_2_O, pH 7.5)) for 30 min before 2 × 5 min washes with TBS-T (0.1% (v/v) Tween20 in TBS-T). Primary antibodies were diluted in antibody buffer (1% (w/v) Milk Powder in TBS-T) and incubated with the membrane overnight at 4 °C. The following antibody dilutions were used: Calcineurin (#2614, CST) 1:1000; Calmodulin (2D1) (ab2860, Abcam) 1:1000; CaMKII alpha (EPR1828) (ab92332, Abcam) 1:1000; GFP (A11122, Invitrogen) 1:10,000; MAP2 (ab32454, Abcam) 1:1000; Neurogranin (AB5620, Millipore Sigma) 1:1000; Phospho-Tau Ser202, Thr205 (AT8) (MN1020, Invitrogen) 1:1000; Phospho-Tau Ser 262 (sc-101813, Santa Cruz Biotechnology) 1:1000; Phospho-Tau Ser396 (PHF13) (#9632, CST) 1:1000; Puromycin (12D10)(MABE343 Millipore Sigma) 1:1000; Tau (Tau46) (#4019, CST) 1:1000; β-Actin (C4) (sc-47778, Santa Cruz Biotechnology); 1:1000. Following incubation, the membrane was washed 2 × 5 min with TBS-T before incubation with the secondary antibodies (Goat anti-Rabbit IgG Peroxidase Conjugated (AP132P, Merck), Gt x Ms IgG (H + L) HRP (AP124P, Merck)), diluted 1:2500 in antibody buffer, for 1 h. Following 2 × 5 min washes with TBS-T and a 10 min wash with TBS, bound antibodies were detected using Amersham ECL TM Western Blotting Detection Reagent (GE Healthcare) or ECL™ Prime Western Blotting System (Cytiva, GERPN2232). Imaging and semi-quantification was undertaken using the Fusion-SL chemiluminescence System (Vilber Lourmat). Original uncropped immunoblots are available in the supplementary information.

### Immunoprecipitation

DIV13 primary neurons were lysed using Triton-X-100 lysis buffer and centrifuged at 2500 × *g* for 20 min at 4 °C. Protein concentration was determined using a BCA assay and equal concentrations were used for immunoprecipitation with GFP-Trap® magnetic agarose beads (Chromotek). Beads were washed 3 × with lysis buffer prior to incubation with lysates for 1 h at 4 °C with constant agitation, followed by a further three washes with lysis buffer. For immunoblotting, samples were denatured in 2× Laemmli buffer and heated to 95 °C for 10 min and the supernatants stored at −20 °C. For LC–MS/MS, the wash buffer was removed after the final wash and the protein-bound beads were kept frozen at −20 °C before use.

### Tandem mass tagging and mass spectrometry

Immuno-isolated samples were shipped on dry ice to the Bristol Proteomics Facility for processing and analysis as described previously [41]. Immunoprecipitates were reduced, alkylated, and digested with trypsin before labelling with tandem mass tag (TMT) ten-plex reagents (Thermo Fisher Scientific). Labelled samples were pooled before high pH RP fractionation. Analysis by nano-LC–MS/MS was undertaken using a synchronous precursor selection and triple-stage mass spectrometry (SPS-MS3) workflow on an Orbitrap Fusion Tribrid mass spectrometer (Thermo Scientific). The raw data files were processed and quantified using Proteome Discoverer software v2.1(PD2.1; Thermo Scientific) and searched against the UniProt Mus musculus database (downloaded February 2021: 55440 entries) plus eGFP-Tau^WT^ and eGFP-Tau^P301L^ sequences using the SEQUEST HT algorithm. Data were normalised against GFP and filtered to satisfy false discovery rate (FDR) of 5%.

Protein groupings were determined by PD2.1. MS data were searched against the human Uniprot database retrieved on 2021-01-14, and updated with additional annotation information on 2021-01-29. Tau protein from both mouse and human species was placed in a single protein group by PD2.1, and peptides unique to human tau and mouse tau were manually identified and summed to give a human-only and mouse-only tau abundance. The protein abundances were Log2 transformed to bring them closer to a normal distribution. Statistical significance was determined using paired *T*-tests. The *p* values were FDR corrected using the Benjamini-Hochberg method. Principle component analysis was calculated using the FactoMineR package and plotted using the ggplot2 package. Principal Components 1 and 2 were plotted to give an indication of the main sources of variance, and 3 and 4 were plotted to infer any further trends.

Protein interaction networks were created using STRING database (v11.5) [42]. Clustering was undertaken using Markov Clustering (MCL) with an inflation parameter of 1.8 as previously recommended for accuracy and separation [43]. Gene ontology (GO) enrichment analysis was undertaken using ClueGO version 2.5.9 [44] with Cytoscape version 3.9.1 [45]. The mass spectrometry proteomics data have been deposited to the ProteomeXchange Consortium via the PRIDE [46] partner repository with the dataset identifier PXD044959.

### Functional validation assays

For surface sensing of translation (SUnSET) puromycin-based assays, primary neurons at DIV13 were treated with 10 μg/ml puromycin for 1 h prior to lysis in Triton-X-100 buffer. Samples were centrifuged at 2500 × *g* for 20 min at 4 °C. For cycloheximide (CHX) treatments, primary neurons were treated with 10 μM cycloheximide 24 h prior to lysis.

### Neurogranin ELISA

The levels of endogenous neurogranin in the media from equal numbers of cortical neurons (3 × 10^5^ cells/well) transduced with either pAAV-hSyn1-eGFP, pAAV-hSyn1-eGFP-Tau^WT^ or pAAV-hSyn1-eGFP-Tau^P301L^ were estimated using the FastScan™ Total Neurogranin sandwich enzyme-linked immunosorbent assay (ELISA) according to the manufacturers protocol (CST). Samples were diluted 1:5 in cell extraction buffer to bring absorbance values into the range of the non-quantitative positive control. Samples were read at 450 nm using an IMark^TM^ microplate reader (BioRad).

### Micro-electrode array

Twenty-four and 48-well CytoView MEA Plates (Axion Biosystems) were coated with 0.1% polyethylenimine (PEI) in borate buffer (100 mM Boric acid, 75 mM NaCl, 25 mM Sodium tetraborate, pH 8.4). 10 μl PEI solution was added to the centre of the well as a droplet and dH_2_O was added to the outer rim of the plate to prevent evaporation. The plates were incubated at 37 °C, 5% CO_2_ for 1 h before washing four times with dH_2_O and being left to airdry overnight.

Primary cortical neurons were prepared as described previously with the addition of laminin (10 μg/ml final concentration) to the cell suspension. Ten microliters cell suspension was added to the centre of the well as a droplet at a density of 12,000 cells/µl and the plates were incubated for 1 h at 37 °C, 5% CO_2_ before the final volume of media was added. Half media changes were undertaken at DIV4 and 8 with complete neurobasal medium supplemented with 1 μg/ml laminin. A further media change was undertaken at DIV11 and then every 3 days with un-supplemented, complete neurobasal medium.

MEA analysis was undertaken using the MaestroPro™ (Axion Biosystems), with conditions being maintained at 37 °C, 5% CO_2_ throughout. Plates were equilibrated in the machine for 5 min prior to recording. Baseline measurements were recorded for 10 min prior to treatment with either 4AP/Bicuculline or DMSO/H_2_O controls. Treatments were delivered by replacing 10% of the media with a 10X stock of 4AP/Bicuculline. Following 10 min of equilibration, treated neurons were recorded for a further 10 min.

The recording data were analysed using the Neural Metric Tool (Axion Biosystems). Network bursts were identified as a minimum of 50 spikes within 100 ms in at least 35% of the electrodes.

## Results

### Overexpression of eGFP-Tau in primary cortical neurons by AAV transduction

**Fig. 1:**
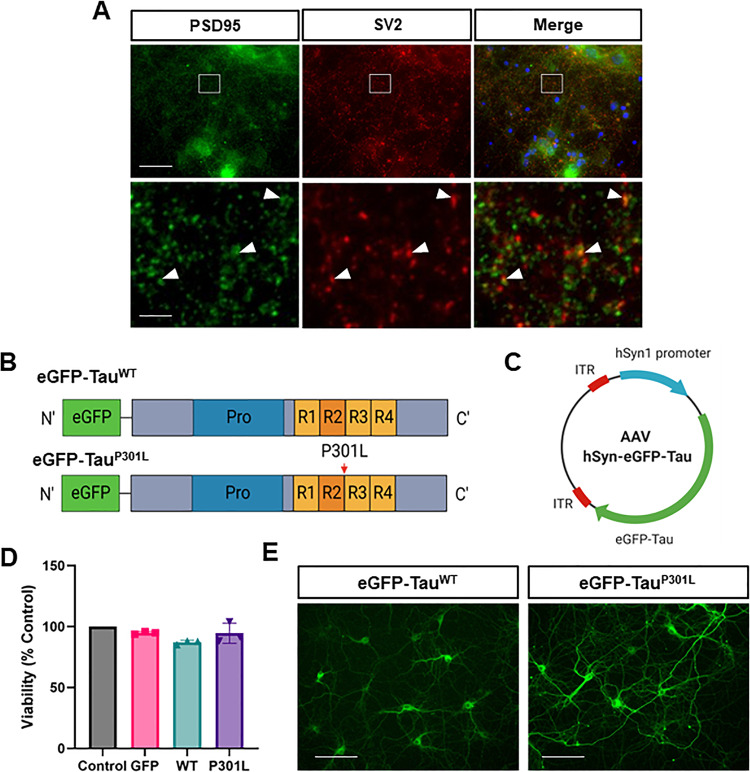
AAV transduction of primary cortical neurons. **A** Primary neurons at DIV14 stained with the pre- and post-synaptic markers SV2 (red) and PSD95 (green) respectively as well as a DAPI stain (blue). Scale bar = 125 µm. Magnified sections show puncta with white arrows highlighting examples of synapses, as shown by overlap of SV2 and PSD-95. Scale bar = 15 µm. **B** The tau constructs used encode wild-type and mutant (P301L) 0N4R human tau, tagged N-terminally with eGFP. Tau has a proline rich region (Pro) and four repeat domains (R1-4). The location of the P301L mutation is shown by the red arrow. **C** Simplified plasmid map of AAV-hSyn-eGFP-Tau: inverted terminal repeats (ITR) are derived from AAV2; the human synapsin 1 (hSyn1) promoter drives neuronal-specific expression of eGFP-Tau. **D** Viability of transduced cultures at DIV14, as measured by MTT assay and presented as the percentage of untransduced control cultures. No significant difference in viability following overexpression of eGFP, eGFP-Tau^WT^ or eGFP-Tau^P301L^ (repeated measures one-way ANOVA with Dunnet’s multiple comparisons test, *n* = 3, *P* > 0.05). Error bars = ± SD. **E** Epifluorescent imaging of DIV14 primary cortical neurons overexpressing eGFP-Tau^WT^ or eGFP-Tau^P301L^.

The emerging view is that pathogenic tau species are synaptotoxic and cause aberrant synaptic excitation, but the underlying molecular mechanisms are unclear [15]. Primary cortical neurons were developed as a platform to model the very earliest stages of tauopathy, before the appearance of secondary effects due to neuronal cell death, as determined by MTT turnover and morphological assessment (see Fig. 1). Primary cortical neurons rapidly formed connected networks, with juxtaposed pre- and post-synaptic markers evident by DIV13 -14 (Fig. 1A). Micro-electrode array analysis demonstrated clear functional connectivity from DIV11 onwards with activity and burst frequency peaking between DIV14-19 and declining thereafter (Supplementary Fig. 1).

**Fig. 2:**
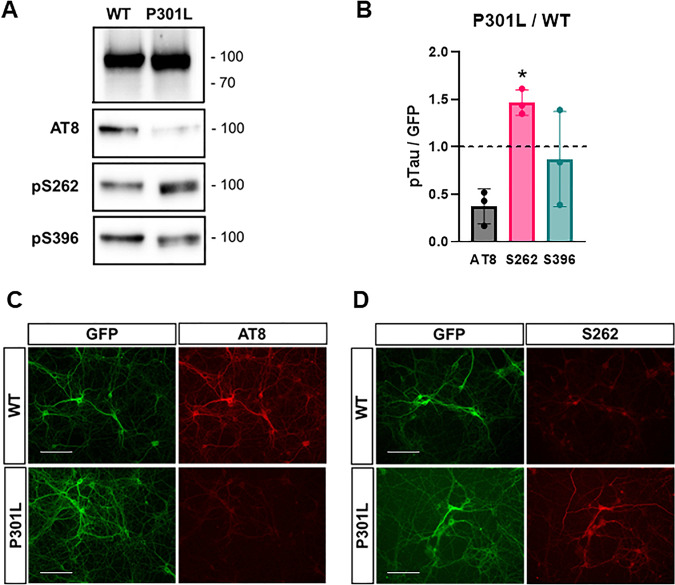
Differential phosphorylation between wild-type and mutant tau. **A** Immunoblot of eGFP-Tau that has been immunoprecipitated by GFP-Trap® and probed with antibodies for different phospho-tau epitopes – AT8 (pSer202, Thr205), pSer262 and pSer396 – as well as GFP, which was used as an estimation of total human tau levels. **B** The levels of eGFP-TauP301L phosphorylation relative to eGFP-TauWT. The dotted line represents an equal amount of the phosphorylation site between both systems. Significant increase in phosphorylation at Ser262 but not AT8 or Ser396 with eGFP-TauP301L (ratio-paired t-test of raw data, *n* = 3, pSer262, *P < 0.05; AT8, *P* = 0.089; pSer396, *P* = 0.549). Error bars = ±SD. Immunofluorescence of transduced DIV13 neurons probed for AT8 (**C**) or Ser262 (**D**) (red). Images taken with the EVOS M7000 imaging system. Scale bar = 100 μm.

To generate an efficient model of tau overexpression, a viral transduction approach was developed using adeno-associated viral particles (AAVs). 0N4R human tau, tagged at the N-terminus with eGFP has shown that tau^P301L^, but not tau^WT^, localised to dendritic spines and caused synaptic dysfunction [21]. Therefore, AAV constructs encoding both eGFP-tagged wild-type and mutant P301L tau under control of the neuronal specific Syn1 promoter were generated (Fig. 1B, C). AAV transduction was undertaken at DIV6, and eGFP expression was clearly detectable from 5 days post viral transduction, equivalent to DIV11 (Supplementary Fig. 2). Experiments were therefore conducted from DIV13, when synapses were judged to be well established. Immunofluorescence microscopy showed a transduction efficiency of ~60% and no detectable expression of eGFP in cells positive for the astrocytic marker GFAP suggesting neuronal specificity (data not shown). No significant differences in viability between untransduced neurons and neurons overexpressing eGFP, eGFP-Tau^WT^ or eGFP-Tau^P301L^ were identified using an MTT assay (Repeated measures one-way ANOVA, *p* = 0.54, *n* = 3) (Fig. 1D). When comparing neurons overexpressing eGFP-Tau^WT^ with eGFP-Tau^P301L^ by live imaging fluorescent microscopy, it was observed that eGFP-Tau^P301L^ appeared at higher levels than eGFP-Tau^WT^ (Fig. 1E), particularly at earlier DIV (Supplementary Fig. 2, also see Figs. 2C; 6C; 7D).

### Differential Tau Phosphorylation Between eGFP-Tau^WT^ and eGFP-Tau^P301L^

To isolate tau, to investigate state-specific phosphorylation, and to identify tau binding partners in primary cortical neurons, affinity purification – mass spectrometry (AP–MS) was employed. To isolate tau and to identify tau binding partners, immunoprecipitation (IP) was undertaken using a GFP-trap®: nanobodies against GFP bound to magnetic beads. Targeting GFP rather than tau enabled the interactomes of eGFP and eGFP-Tau to be directly compared, ensuring that defined binding partners were specific to tau rather than the tag and eliminated potentially confounding effects of endogenous tau.

The phosphorylation status of tau immunoprecipitated for AP-MS was defined. Three phospho-epitopes were examined: AT8 (pSer202/pThr205) – a phosphoepitope associated with tau aggregation as well as tau accumulation at the post-synaptic compartment [16, 19, 47, 48]; Ser262 – one of the earliest and most significantly changed phosphorylation sites in AD that hinders the association of tau with microtubules [49, 50]; and Ser396 – phosphorylation of which promotes tau mislocalization to the post-synaptic compartment [51] (Fig. 2A, B). Immunoblotting with phosphorylation-state-specific antibodies showed that phosphorylation at Ser396 was unchanged although variable between tau^WT^ and tau^P301L^. Given that Ser396 is a C-terminal residue, and tau measured here was isolated with a GFP-trap®, it was considered that some phosphorylated C-terminal fragments might not be detected. Therefore, phosphorylation at Ser396 was also measured in total cell lysates and measured against Tau46, which targets the C-terminus of tau, rather than GFP. However, similar to the immunoprecipitation experiments, Ser396 phosphorylation was again variable (Ratio-paired *t*-test, *p* = 0.549) with no consistent difference in phosphorylation between tau^WT^ and tauP^301L^ (Supplementary Fig. 3A–C) In contrast, immunoprecipitated tau^P301L^ exhibited significantly higher levels of Ser262 phosphorylation (Ratio-paired *t*-test, *p* < 0.05, *n* = 3) (Fig. 2A, B). AT8 phosphorylation with immunoprecipitated tau^P301L^ was decreased by more than 50% compared to tau^WT^, although this failed to reach statistical significance (Ratio-paired *t*-test, *p* = 0.089). Qualitative, immunofluorescence imaging confirmed the same differential phosphorylation pattern of AT8 and Ser262 between tau^WT^ and tau^P301L^ (Fig. 2C, D). Phosphorylation at Ser217, only 12 residues C-terminal to AT8 was also decreased in eGFP-Tau^P301L^ relative to eGFP-Tau^WT^ (Supplementary Fig. 3D, E). Thus, differential patterns of phosphorylation between eGFP-Tau^P301L^ relative to eGFP-Tau^WT^ and the apparent somatodendritic expression were together suggestive of dysregulation following overexpression of mutant and wild-type tau within neurons. To explore potential mechanisms at the level of binding partners, the neuronal tau interactome was defined.

### The human tau interactome in primary cortical neurons

**Fig. 3:**
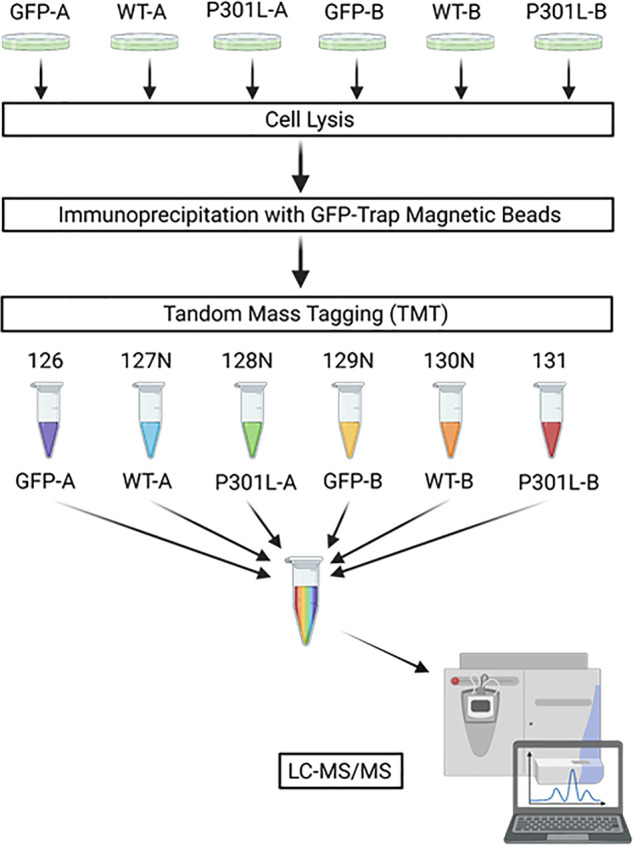
Workflow for determining the tau interactome by AP-MS. **A** DIV13 primary neurons, transduced with AAV’s to express eGFP, eGFP-Tau^WT^ or eGFP-Tau^P301L^ were lysed in 1% Triton-X-100 lysis buffer and the cell lysates were immunoprecipitated with GFP-Trap® magnetic beads. The samples were digested on the beads and then labelled with TMT reagents and pooled before analysis by LC–MS/MS. Figure created with BioRender.com.

For interactomics, biological replicates of DIV13 neurons prepared from two independent transduced cultures, expressing either eGFP, eGFP-Tau^WT^ or eGFP-Tau^P301L^ were processed for mass spectrometry (Fig. 3). GFP-Trap® IP, 6-Plex tandem-mass-tagging (TMT) was then undertaken to enable quantitative comparisons of the interactomes.

Principle component analysis showed that the greatest source of variation was between the eGFP and eGFP-Tau interactomes, suggesting that tau has a greater influence on the interactome than the eGFP-tag (data not shown). To further ensure that candidate interactors were specific to tau rather than the eGFP-tag, the threshold for eGFP-specific binding was set at log fold-change <1.5 between eGFP and eGFP-Tau, as has been used in similar studies [36]. In total, 585 candidate interactors were identified by LC–MS/MS, 96 of which lacked the quantitative information necessary to rule out eGFP-specific binding. However, all 489 remaining candidate interactors (Supplementary Table 1 and Supplementary Data Set) reached the threshold for tau-specific binding (>1.5 log2 fold change greater association with eGFP-Tau than eGFP) and summarised in Supplementary Table 1.

**Fig. 4:**
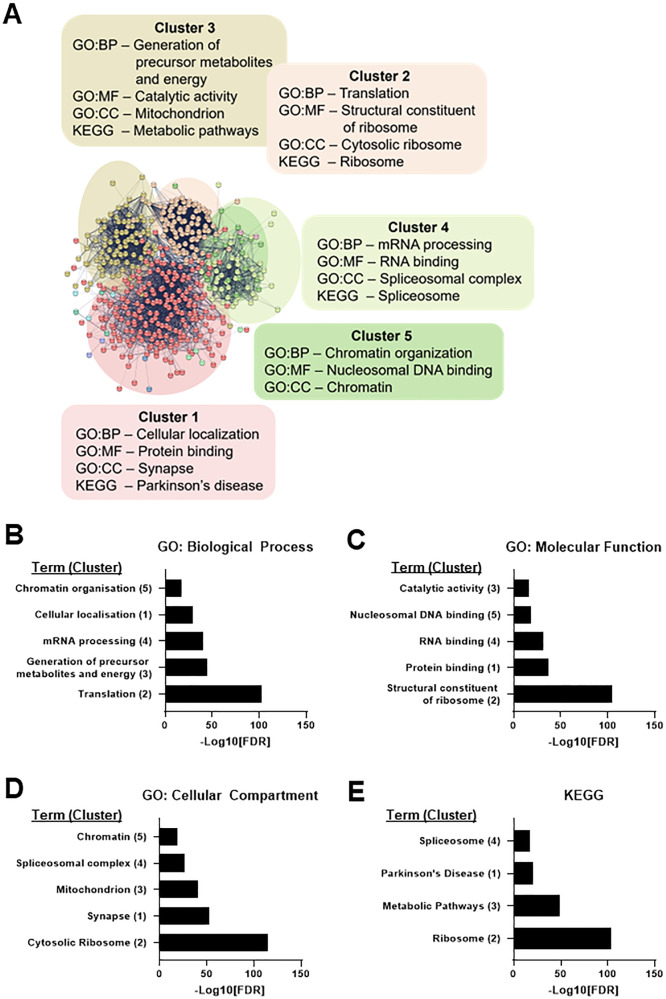
The tau^WT^ interactome in primary cortical neurons. **A** The STRING interaction network, incorporating both functional and physical protein associations, of 481 proteins identified as candidate tau interactors by AP–MS. Clusters were formed by MCL clustering (MCL inflation parameter = 1.8) of the interaction network. 11 clusters in total were identified, the five largest clusters are highlighted. Functional enrichment analysis of each cluster was undertaken for GO: Biological Process (BP), GO: Molecular Function (MF), GO: Cellular Compartment (CC) and KEGG. The most significant descriptor (assessed as the term with the smallest false discovery rate (FDR)) for each enrichment category is listed unless it was irrelevant to neurons, in which case the next most significant descriptor was included. The FDR for the top term in each cluster for each sub-ontology (GO:BP (**B**), GO:MF (**C**), GP:CC (**D**), KEGG (**E**)) is shown in order of significance, with cluster number in brackets next to the term, highlighting that eGFP-Tau^WT^ is most significantly enriched with proteins associated with the ribosome and translation (cluster 2). Both clustering and functional enrichment analysis were undertaken in STRING-db (v11.5). GO Gene Ontology, KEGG Kyoto encyclopaedia of genes and genomes.

To explore which pathways or processes associated with wild-type tau, gene-ontology (GO) enrichment analysis was undertaken for the eGFP-Tau^WT^ interactome. Markov clustering of the eGFP-Tau^WT^ interactome dataset using the STRING database identified eleven functional clusters, of which the five largest are highlighted (Fig. 4A). Enrichment analysis was undertaken on these clusters, incorporating GO terms for biological function, molecular processes, cellular compartment as well as KEGG (Kyoto encyclopaedia of genes and genomes). This analysis revealed that the eGFP-Tau^WT^ interactome was most significantly enriched for ribosomal proteins and proteins involved in translation (Cluster 2) (Fig. 4B–E). Furthermore, significant enrichment for RNA binding proteins (Cluster 4) as well as chromatin associated proteins (Cluster 5) further supports a role for tau in gene expression. There was also notable enrichment for mitochondrial proteins (Cluster 3). Importantly, “Synapse” was the term most enriched in Cluster 1 for cellular compartment analysis (Fig. 4D), highlighting the importance of studying the tau interactome in cellular models which form functional synapses.

### Differences in synaptic, ribosomal and proteasomal associations between wild-type and mutant tau

**Fig. 5:**
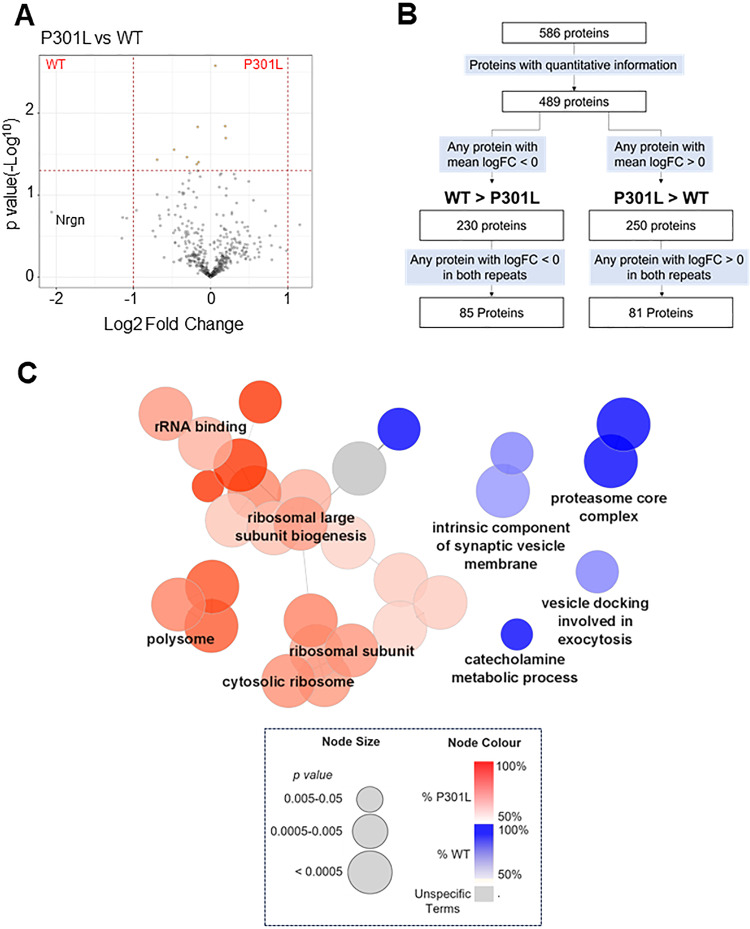
Comparison of eGFP-Tau^WT^ and eGFP-Tau^P301L^ interactomes. **A** A volcano plot of the Log2 Fold Change (FC) versus the *p*-value (-Log10; t-test, *n* = 2) representing the quantitative differences in the tau interactome between eGFP-Tau^WT^ and eGFP-Tau^P301L^. Log2FC > 0 represents proteins more associated with eGFP-Tau^P301L^, whereas Log2FC < 0 represents proteins more associated with eGFP-Tau^WT^. The horizontal red dashed line marks the threshold for significance while the vertical red dashed lines mark -1 and +1 Log2FC. Yellow circles represent significantly different proteins. The largest fold change was seen with neurogranin (Nrgn) which is labelled. **B** Proteins were systematically filtered based on a series of criteria to produce two groups, with proteins consistently more associated to either eGFP-tau^WT^ or eGFP-Tau^P301L^. **C** A summary of ClueGO-based enrichment analysis of candidate interactors showing preference for either eGFP-Tau^WT^ and eGFP-Tau^P301L^. The colour of the node represents the relative enrichment in each group: blue represents terms enriched in eGFP-Tau^WT^ group; red represents terms enriched in eGFP-Tau^P301L^ group; and grey represents terms that exhibit similar levels of enrichment for both groups. 100% enrichment for P301L would mean that all the proteins within that term that have been identified as tau candidate interactors are more associated with eGFP-Tau^P301L^ than eGFP-Tau^WT^. The size of the node relates to the *p*-value, with larger nodes representing greater statistical significance. GO: Cellular Compartment, GO: Molecular Function and GO: Biological Processes were analysed.

In the eGFP-Tau^P301L^ interactome dataset, only the mitochondrial protein ATP synthase F1 subunit delta (Atp5f1d) did not reach the threshold for tau-specific binding (eGFP-Tau^P301L^/eGFP: log_2_FC = 1.4). The greatest difference in association between eGFP-Tau^WT^ and eGFP-Tau^P301L^ was neurogranin, which was more than twofold more associated with eGFP-Tau^WT^ than eGFP-Tau^P301L^ (Fig. 5A). Neurogranin is a post-synaptic protein that binds to calmodulin in the absence of Ca^2+^, modulating synaptic signalling and strength, possibly by regulating the localisation of calmodulin [52–56]. Further investigation revealed that calmodulin and the binding partners Ca^2+^/calmodulin-dependent kinase II (CaMKII), calcineurin and neuromodulin (GAP43) were candidate interactors of tau. eGFP-Tau^P301L^ was more and less associated with CaMKII-α (Camkiia; log_2_FC = 0.37) and calcineurin (Ppp3ca; log_2_FC = −0.47; t-test, *p* = 0.028) than eGFP-Tau^WT^, respectively suggesting that tau is involved in the co-ordination of calcium/calmodulin signalling at the synapse which may be disrupted by Tau^P301L^. A full summary list of differentially associated interactors is provided (Supplementary Table 1).

When comparing the wild-type and mutant tau interactomes, only proteins in which both repeats exhibited a logFC less than zero or greater than zero were included in enrichment analysis (Fig. 5B). This revealed that eGFP-Tau^P301L^ was more associated with ribosomal and translational proteins, but less associated with the proteasome core complex (Fig. 5C).

The increased association with ribosomal proteins could reflect an impairment in translation, and to explore this a puromycin-based assay was undertaken. Puromycin is a tyrosyl-tRNA mimic that labels polypeptide chains as they are translated and releases them from the ribosome [57] (Supplementary Fig. 4A). Monoclonal antibodies against puromycin were used to detect newly synthesised proteins and thus indirectly assess the level of active translation. The level of active translation was consistently reduced in cells overexpressing eGFP-Tau^P301L^ than eGFP or eGFP-Tau^WT^, although this only reached statistical significance when compared to eGFP-Tau^WT^ (repeated measures one-way ANOVA with Tukey’s multiple comparisons test, *P* = 0.12, *n* = 3) (Supplementary Fig. 4B).

**Fig. 6:**
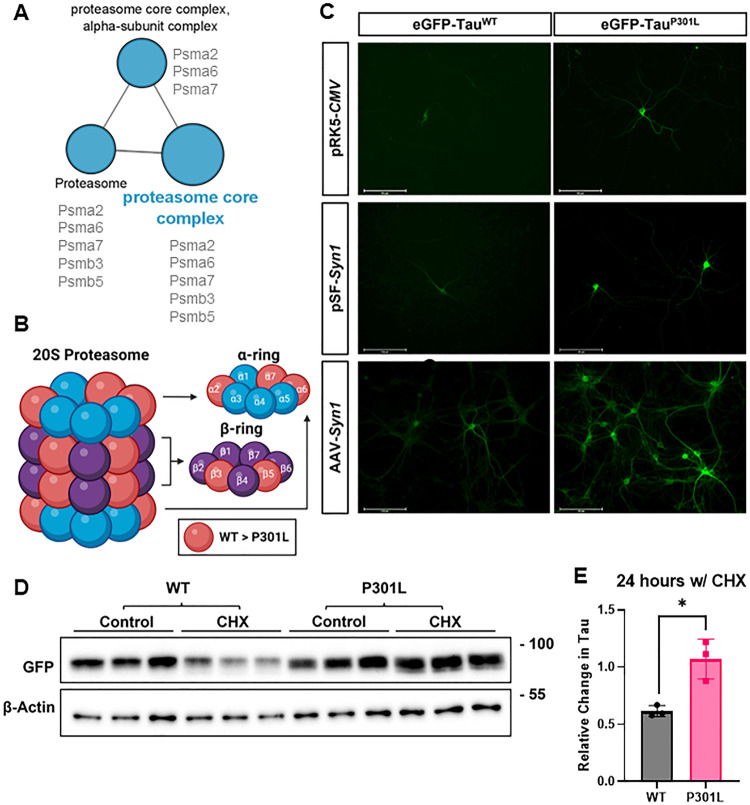
Decreased association of eGFP-Tau^P301L^ with the proteasome core complex correlates with impaired htau degradation. **A** eGFP-Tau^P301L^ was less associated with the proteasome core complex 20S subunits alpha 2/6/7 (Psma2/6/7) and beta 3/5 (Psmb3/5) than eGFP-tau^WT^. **B** These subunits are constituents of the alpha and beta rings of the 20S proteasome core complex – subunits less associated with eGFP-Tau^P301L^ are coloured red. Figure created with BioRender.com. **C** Increased levels of eGFP-Tau^P301L^ relative to eGFP-Tau^WT^ have been observed across multiple iterations of the model as demonstrated by representative fluorescent microscopy images of neurons following lipofectamine2000™ based transfection of pRK5-*CMV*-eGFP-Tau and pSF-hSyn1-eGFP-Tau as well as AAV-based transduction of AAV-hSyn1-eGFP-Tau, DIV13, Scale bar = 125 µm. **D**, **E** DIV13 primary cortical neurons transduced with AAV-Syn1-eGFP-Tau^WT^ or AAV-Syn1-eGFP-Tau^P301L^ were incubated with 10 μM cycloheximide (CHX) for 24 hours to inhibit protein synthesis, controls were lysed at the same timepoint without treatment. **D** Immunoblot of corresponding lysates (three independent treatments) probed for GFP as marker of total htau or β-actin (loading control). **E** The relative change in tau levels following 24 h treatment with CHX (w/CHX) compared with non-treated control (set as 1.0). eGFP-Tau^WT^ levels (grey bar) decreased significantly more than eGFP-Tau^P301L^ levels (pink bar; *t*-test, *p < 0.05, *n* = 3 biological repeats (three technical repeats per biological repeat). Error bars = ±SD.

The decreased association of eGFP-Tau^P301L^ with the proteasome core complex could indicate an impairment in proteasomal degradation of tau (Fig. 6). eGFP-Tau^P301L^ was consistently present at higher levels than eGFP-Tau^WT^, both in the AAV transduction model and when transfected by lipofectamine 2000^TM^ under the control of either hSyn1 or the ubiquitous CMV promoter: which could be explained by impaired tau degradation (Fig. 6C). Furthermore, impaired degradation of the P301L tau mutant has been shown previously using cycloheximide (CHX) chase assays in SH-SY5Y cells and brain slices from transgenic mice [58]. To investigate this, protein synthesis was inhibited with CHX and the level of protein degradation over time was assessed by comparison with untreated cells. After 24 h treatment with CHX, the levels of eGFP-Tau^WT^, were reduced by ~50% (Fig. 6D, E). In contrast, the levels of eGFP-Tau^P301L^ in CHX did not differ significantly from untreated cells suggesting that eGFP-Tau^P301L^ degradation is likely impaired relative to eGFP-Tau^WT^.

### Overexpression of eGFP-Tau^P301L^ does not influence the levels of calmodulin signalling proteins but releases neurogranin

**Fig. 7:**
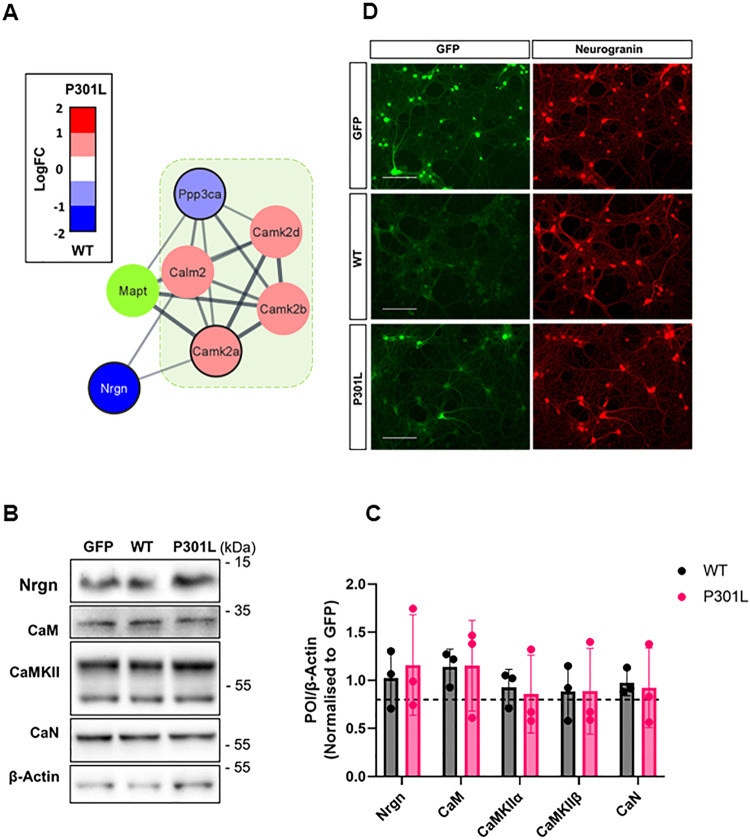
eGFP-TauP301L expression does not change the total levels of neurogranin or other components of the calmodulin-binding module. **A** STRING interaction network of calmodulin-binding proteins. Node colours represent preference for tauWT (blue) or tauP301L (red/pink) and are scaled relative to LogFC (P301L/WT). Nodes with black outline represent proteins that showed same binding preference in both replicates. Direct binding partners of tau are highlighted in green overlay. Ppp3a (Calcineurin); Calm2 (Calmodulin); Camk2a/b/d (CamKII); Nrgn (Neurogranin); Mapt (Tau). **B** Immunoblot of neuronal lysates (DIV13) from eGFP (GFP), eGFP-Tau^WT^ (WT) and eGFPTau^P301L^ (P301L) expressing cells. Blots probed with antibodies against neurogranin (Nrgn); calmodulin (CaM); Ca2+/CaM-dependent kinase II (CamKII) (Upper band CaMKIIβ; lower band CamKIIα), calcineurin (CaN), or β-Actin. The position of molecular weight markers (kDa) indicated to right. Blot representative example of *n* = 3. **C** Semi-quantitation (ECL) of immunoreactive bands from the proteins of interest (POI) normalised first against the β-Actin loading control. Values from eGFP-Tau^WT^ (grey bars) and eGFP^P301L^ (pink bars) were then normalised to eGFP samples (represented by the dotted line). No significant differences were found in the levels of any POIs between eGFP-Tau^WT^ and eGFPTau^P301L^ (*T*-test, *n* = 3). **D** Immunofluorescence imaging of DIV13 primary neurons transduced with eGFP, eGFP-Tau^WT^ or eGFP-Tau^P301L^ (green) and probed with an antibody against neurogranin (red) Scale bar = 125 µm showing neurogranin is localised to the cell body and neurites.

The differential association of calmodulin signalling proteins between wild-type and mutant tau (Fig. 7A and see Supplementary Table 1) could potentially be explained by changes in expression levels, so the overall protein levels of these interactors were determined. Immunoblotting detected bands of the expected molecular weights for neurogranin, calmodulin, CamKII, and calcineurin but there were no measurable differences in the levels of these proteins between tau^WT^ and tau^P301L^ expressing neurons relative to β-actin (Fig. 7B, C). Furthermore, there was no clear difference in neurogranin levels or subcellular localisation of neurogranin between tau^WT^ and tau^P301L^ expressing neurons as determined by immunofluorescence staining (Fig. 7D). Despite this, increased levels of neurogranin were detected in the media harvested from equal numbers of Tau^P301L^ expressing cells compared with tau^WT^ cells (Supplementary Fig. 5). This suggests that following disengagement from tau^P301L^ a pool of neurogranin is released or potentially leaks from neurons and this occurs in the absence of obvious changes in the total levels of neurogranin or any clear morphological metric of neurotoxicity.

### Overexpression of eGFP-Tau^P301L^ Causes Basal Hyperexcitability

**Fig. 8:**
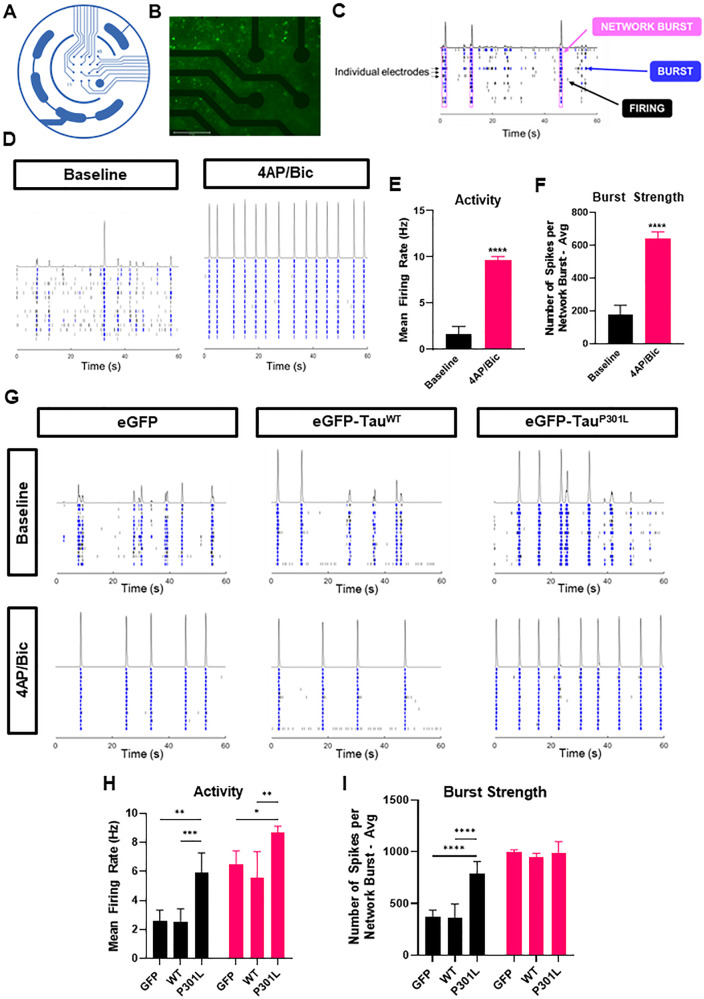
Network analysis with micro-electrode arrays demonstrates basal hyperexcitability in neurons overexpressing eGFP-Tau^P301L^. All experiments were undertaken in DIV12 cultures cultured in CytoView micro-electrode array (MEA) multi-well plates using the Maestro-Pro MEA system (Axion Biosystems). **A** Each well contains 16 electrodes that can record local activity **B** Fluorescent microscopy shows DIV12 primary cortical neurons transduced with AAV-Syn1-eGFP and cultured on a micro-electrode plate. Scale bar = 275 µm. **C** An example of a raster plot, which shows firing over time, taken at baseline from untransduced neurons. Each row corresponds to an individual electrode. Action potentials are recorded as spikes (black lines) and this is termed ‘firing’. A burst (blue) occurs when multiples action potentials fire in rapid succession in the locality of an electrode; a minimum threshold is set at 50 spikes within 100 ms. A network burst was classified as at least 35% of electrodes recording bursts simultaneously; this shows that the neurons throughout the culture are connected. **D** Representative raster plots showing 60 s recordings of untransduced neurons at baseline and following treatment with 0.625 µM 4AP/12.5 µM Bicuculline (Bic). Baseline recordings show spontaneous firing and bursting. Treatment with 4AP/Bic stimulates synchronous and oscillatory firing across the well, as shown by a switch to network bursting. **E** 4AP/Bic treatment significantly increases activity and (**F**) burst strength (*t*-test, ****P < 0.0001, *n* = 3 technical repeats). **G** Representative raster plots comparing neurons overexpressing eGFP, eGFP-Tau^WT^ or eGFP-Tau^P301L^ at baseline and following treatment with 4AP/Bic. **H** At baseline, the activity and (**I**) burst strength of neurons overexpressing eGFP-Tau^P301L^ is significantly greater than those expressing eGFP or eGFP-Tau^WT^. Following treatment with 4AP/Bic, activity but not burst strength is significantly greater with eGFP-Tau^P301L^ than eGFP or eGFP-Tau^WT^ (two-way ANOVA with Tukey’s multiple comparisons test, *p < 0.05, **p < 0.01, ***p < 0.001, ****p < 0.0001, *n* = 4 technical repeats).

The differential effects on calmodulin binding partners implicated likely changes at the levels of calcium signalling and neurotransmission so the effects of tau expression on neuronal activity were investigated. This was undertaken using a multi-well micro-electrode array (mwMEA) platform which enables non-invasive measurement of neuronal activity at a network level [59, 60]. Each well of the multi-well plate contains 16 electrodes that can detect local action potentials (Fig. 8A, B). Multiple action potentials in quick succession suggests local connectivity and is classified as a burst while synchronous bursting across electrodes suggests network connectivity throughout the well (Fig. 8C). At DIV13, primary cortical neurons exhibited spontaneous firing and bursting, confirming that the neurons were electrically active and had formed functional synapses (Fig. 8D and see Supplementary Fig. 1). Stimulation with the voltage-gated potassium channel antagonist 4AP and the GABA_A_ receptor antagonist bicuculline induced oscillatory network bursting and significantly increased the firing rate and burst strength (Fig. 8D–F).

Comparison of baseline recordings from transduced cultures demonstrated an increase in basal excitability with overexpression of eGFP-Tau^P301L^ relative to eGFP or eGFP-Tau^WT^ (Fig. 8G–I). More specifically, there was a significant increase in activity and burst strength, with more than twice the mean firing rate and number of spikes per network burst than eGFP or eGFP-Tau^WT^. An independent set of experiments spanning DIV13 - DIV20 to take into account possible developmental changes showed that patterns of enhanced excitability following eGFP-Tau^P301L^ were maintained across the time-course (Supplementary Fig. 6). Interestingly, overexpression of eGFP-Tau^WT^ had little effect on any of the parameters measured compared to the eGFP control, suggesting that overexpression of wild-type tau alone is not sufficient to enhance network activity at these time points.

As with untransduced neurons, stimulation with 4AP/Bicuculline significantly increased activity (Šidáks multiple comparisons test: GFP, *P* = 0.0002; WT, *P* = 0. 0.0044; P301L, *P* = 0.0055) and burst strength (Šidáks multiple comparisons test; GFP: *P* < 0.0001; WT: *P* < 0.0001; P301L: *P* = 0.02) of all groups (Fig. 8G-I). However, the relative increase in burst strength with stimulation was significantly smaller in neurons overexpressing eGFP-Tau^P301L^ compared with that seen with eGFP-Tau^WT^ (one-way ANOVA with Tukey’s multiple comparisons test: GFP vs P301L, *P* = 0.013; WT vs P301L, *P* = 0.036), with no significant difference in burst strength between groups once stimulated. This suggests that eGFP-Tau^P301L^ overexpression increases basal excitability but may attenuate the distinction between unstimulated and stimulated states which is necessary for synaptic plasticity.

## Discussion

Comparative analysis of mouse primary cortical neurons transduced with AAVs expressing eGFP, eGFP-Tau^WT^ and eGFP-Tau^P301L^, demonstrated differences in tau phosphorylation, the tau interactome and neuronal network activity. The presence of the P301L mutation was associated with enhanced phosphorylation at Ser262 and stabilisation of tau, likely through disassociation from components of the proteasomal core complex. Notably, the P301L mutation also caused a clear increase in basal network excitability, and this correlated with increased association with CamKII and decreased association with calcineurin and neurogranin compared with tau^WT^. The disengagement of tau from neurogranin could explain its subsequent appearance as a sensitive CSF disease biomarker and the high extracellular levels of neurogranin in tau^P301L^ culture media is consistent with this. Collectively, this gives insight into some of the earliest changes in neuronal function driven by tau^P301L^ which might underpin resultant synaptotoxicity.

It was notable that neurons overexpressing tau^P301L^ appeared more synaptically active than tau^WT^, which could be linked to the spine enrichment reported in young P301L mice [61] and provides a context for differential phosphorylation. A difference in phosphorylation at Ser262 between tau^WT^ and tau^P301L^ in isolated neurons has not previously been described. Here, we have shown that tau^P301L^ Ser262 phosphorylation was increased compared to tau^WT^ which could impact the function and stability of tau, as Ser262 phosphorylation reduces the affinity of tau for microtubules and impairs proteasomal degradation of tau [49, 62–64]. Furthermore, the Ser262 phospho-epitope has been identified as one of the earliest post-transcriptional modifications in AD [50], therefore, specific increases in Ser262 phosphorylation in the absence of more general hyperphosphorylation suggests that this could be an early change associated with synaptic hyperactivity at the onset of tau pathology. The increase in pSer262 levels with tau^P301L^ might relate to the parallel increased association with the kinase CaMKII and decreased association with the phosphatase calcineurin – both of which act at the Ser262 phospho-epitope [65–67]. However, this needs to be confirmed, particularly given the absence of a key Ser262 kinase, MARK, from the identified tau interactome [68, 69]. The absence of MARK as well as other tau kinases such as CDK5 and Fyn is likely due to the transient nature of these interactions which are harder to capture by AP-MS.

Unexpectedly, AT8 phosphorylation of eGFP-Tau^P301L^ was decreased, relative to eGFP-Tau^WT^. It is possible that this difference reflects increased AT8 phosphorylation with tau^WT^, however, this is not the first instance in which phosphorylation at AT8, which is associated with an increased propensity for aggregation [47], has been found to be lower with the P301L mutant, as this has a been observed in mice where 2N4R tau^WT^ was more phosphorylated than tau^P301L^ [70]. In contrast, phosphorylation at AT8 was found to be increased in rat primary hippocampal neurons overexpressing eGFP-Tau^P301L^ relative to eGFP-Tau^WT^ [22] and in vitro studies reported that tau^P301L^ is phosphorylated to a greater extent, and at a faster rate, than tau^WT^ when incubated with rat brain extract [71]. These inconsistencies could relate to species differences, or differences in the age of the neuronal cultures although this would not easily explain the previous in vivo findings [70, 72]. The immunoblotting findings were confirmed by immunofluorescence, suggesting that the decreased AT8 phosphorylation was probably not due to a loss of highly phosphorylated insoluble tau aggregates in the preparation of soluble lysates although this was not determined. Phosphorylation at Ser217 appeared to be similarly decreased with the P301L mutation. The close proximity of Ser217 to AT8 (Ser202/Thr205) could indicate the presence of local conformational changes reducing phosphorylation within this domain. The functional consequences of Ser217 tau phosphorylation are not known, however, increased levels of plasma pSer217 tau is a promising blood biomarker for AD [73, 74].

Of the 489 candidate interactors identified by quantitative interactors, 90% (440 proteins) had been identified previously [75]. Of these, only three were specific to a mouse background while 39% (170 proteins) had only been detected on a human background prior to this study. The enrichment of tau with ribosomal and RNA-binding proteins is typically seen as a feature of the human, but not rodent, tau interactome [75]. Our findings contradict this, as ribosomal proteins were the most significantly enriched in this dataset.

Enrichment analysis of eGFP-Tau^P301L^ vs eGFP-Tau^WT^ interactomes revealed several similarities to a previous interactomics study undertaken with C-terminally tagged tau expressed in SH-SY5Y cells [35]. Most notably, the increased enrichment of tau^P301L^ for ribosomal proteins and decreased association with proteasomal proteins. It is important to note that affinity purification of tau overexpressed in human-derived iPSC neurons showed a decreased association of tau^P301L^ with ribosomal proteins compared with tau^WT 36^. However, due to the absence of isobaric labelling for accurate quantitative comparison, a high threshold was necessary for inclusion and so more subtle differences may have been missed that were observed here.

Preliminary experiments to explore effects of eGFP-Tau^P301L^ on translation suggested that eGFP-Tau^P301L^ impaired general translation relative to eGFP-Tau^WT^. This is in-line with previous studies both in vitro and in vivo which have demonstrated impairment of translation by mutant tau [12–14, 76]. The decreased association of eGFP-Tau^P301L^ with the 20S proteasome relative to eGFP-Tau^WT^ could reflect a decrease in proteasomal degradation of tau^P301L^ and a CHX-chase assay confirmed an impairment in tau degradation. This has been reported previously, with the peptidyl-prolyl cis/trans isomerase (Pin1) suggested to differentially regulate WT and mutant tau stability [58]. Pin1 was not identified by mass spectrometry as a candidate tau interactor in this study, although this does not rule out association. The impaired degradation of eGFP-Tau^P301L^ observed here could relate to increased phosphorylation at Ser262. Phosphorylation of tau by CaMKII at Ser262 as well as Ser324, Ser252 and Ser356 inhibits tau degradation by the 20S proteasome [77]. Furthermore, phosphorylation at Ser262/Ser356 increased tau stability in CHO cells stably expressing 0N4R tau^P301L^ and prevented ubiquitination and degradation by the E3 ubiquitin ligase CHIP [64, 78].

Differences in the association of mutant tau with Ca^2+^/calmodulin binding proteins implicates disruption to synaptic signalling. The decreased association of eGFP-Tau^P301L^ with neurogranin is particularly intriguing as neurogranin is a potential biomarker of synapse dysfunction in AD [79, 80]. It was recently shown that in AD the levels of neurogranin in the synapse decreased prior to synaptic loss, indicating that the loss of neurogranin is not simply a correlate of synapse loss [81]. Therefore, the decreased association of neurogranin with eGFP-Tau^P301L^ could reflect early changes in synaptic function. Neurogranin has been identified previously in tau-interactomic studies but only on a mouse background, therefore, further experiments in human-derived cells, such as iPSCs, are needed [82, 83].

Micro-electrode array analysis confirmed that the primary cortical cultures exhibited spontaneous network activity responsive to low concentrations of 4AP/Bic. Comparison of transduced neurons revealed a striking increase in basal excitability with eGFP-Tau^P301L^ overexpression. This agrees with previous findings suggesting that tau^P301L^ overexpression promotes basal excitability, as demonstrated by brain slice electrophysiology of transgenic vs non-transgenic mice. For example, hippocampal slices from the Tau^P301L^ mouse model exhibited increased basal synaptic transmission [84] while cortical slices from rTg4510 mice exhibited a more depolarised resting membrane potential at baseline and an increase in evoked action potentials [85, 86]. Similar findings have been observed by introducing oligomeric tau into CA1 pyramidal cells in hippocampal slices, which caused significant depolarisation of the resting membrane potential and increased the frequency of action potentials [87]. However, in contrast, in vivo electrode recordings and in vivo two-photon Ca^2+^ imaging have suggested that tau^P301L^ overexpression suppresses excitability, suggesting that overall effects on hyperexcitability are complex and context dependent [88–90]. The underlying explanation for enhanced excitability is unclear but could reflect alterations in the maturation kinetics of the network. Preventing aberrant excitability through enhanced clearance of tau or through stabilisation of synaptic neurogranin and other calmodulin-binding partners is a potential therapeutic strategy for tau-driven neurodegeneration.

### Supplementary information

Supplementary Figs 1-6

Proteomics summary list of differentially associated interactors

Full Proteomics Data Set

Uncropped Western blots

## Data Availability

The mass spectrometry proteomics data have been deposited to the ProteomeXchange Consortium via the PRIDE partner repository with the dataset identifier PXD044959. Supporting proteomics data and original immunoblots are also available in the Supplementary files.
