# Supplementary material for: TauP301L disengages from the proteosome core complex and neurogranin coincident with enhanced neuronal network excitability
Source: Cell Death Dis. 2024 Jun 18;15(6):429. doi: 10.1038/s41419-024-06815-2 (PMC11189525; doi:10.1038/s41419-024-06815-2)
Supplement: Supplementary file 1 — Supplementary Figs 1-6 [file 41419_2024_6815_MOESM1_ESM.docx]

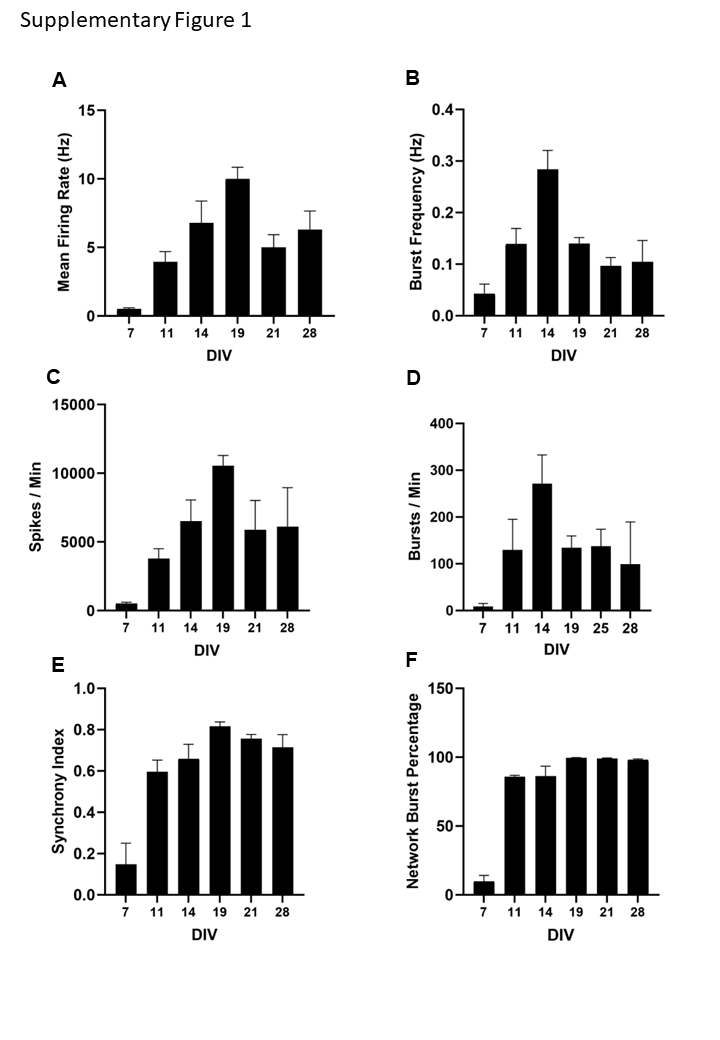


Supplementary Figure 1. Development of the baseline neuronal network in non-transduced primary cortical neurons. Neurons were cultured in CytoView micro-electrode array (MEA) multiwell plates for 7-28 days in vitro (DIV) and monitored for the appearance of spontaneous network activity using the Maestro-Pro MEA system (Axion Biosystems). Recordings were made for 2 min from 5 independent wells of cells at each time-point shown. Data is presented as A) Mean Firing rate B) Burst Frequency C) Spikes per Minute (Spikes / Min) D) Bursts per Minute (Bursts / Min) E) Synchrony Index F) Network Burst Percentage. Data is mean +/- SD n = 5 technical repeats.


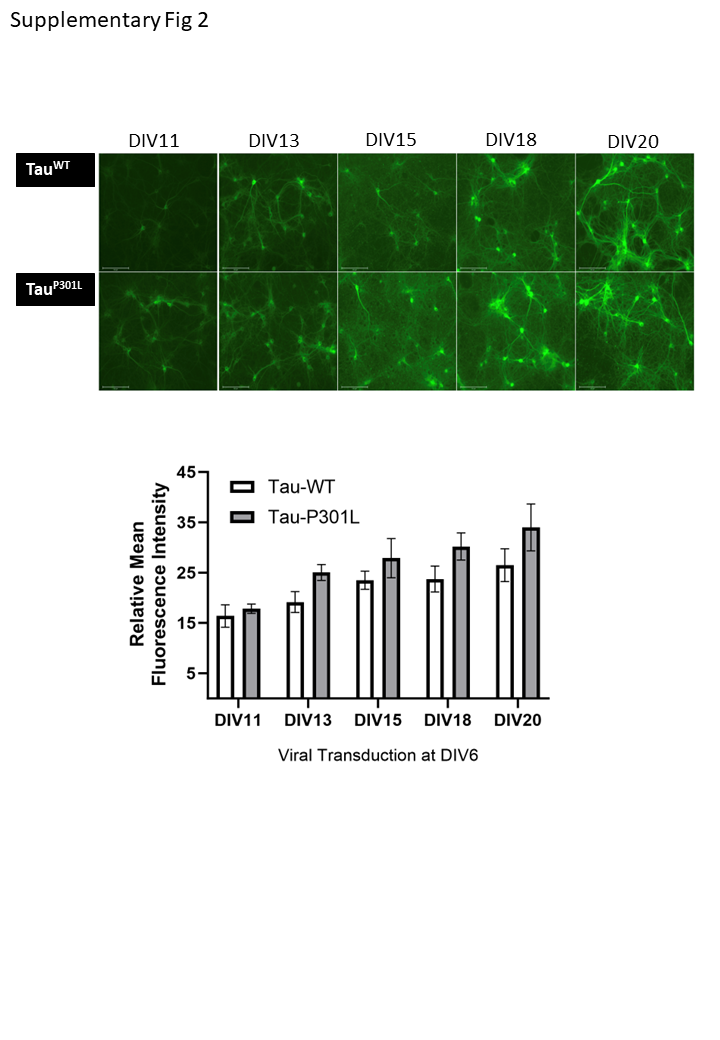


Supplementary Figure 2. Developmental profile of Tau-GFP expression following viral transduction. Primary cortical neurons were cultured in Nunc™ multi-well dishes and transduced at DIV6 with AAV-hSyn1-eGFP-TauWT (TauWT) upper panels or AAV-hSyn1-eGFP-TauP301L (TauP301L) lower panels. GFP fluorescence was monitored in the same transduced cells from DIV11 to DIV20 using an EVOS™ M7000 imaging system. Scale bar 200µM. Mean fluorescence intensity was quantified using Image J. Data shows relative mean fluorescence intensity +/- SD captured from 5 randomly selected fields for each well, with 3 technical repeats for each time point.


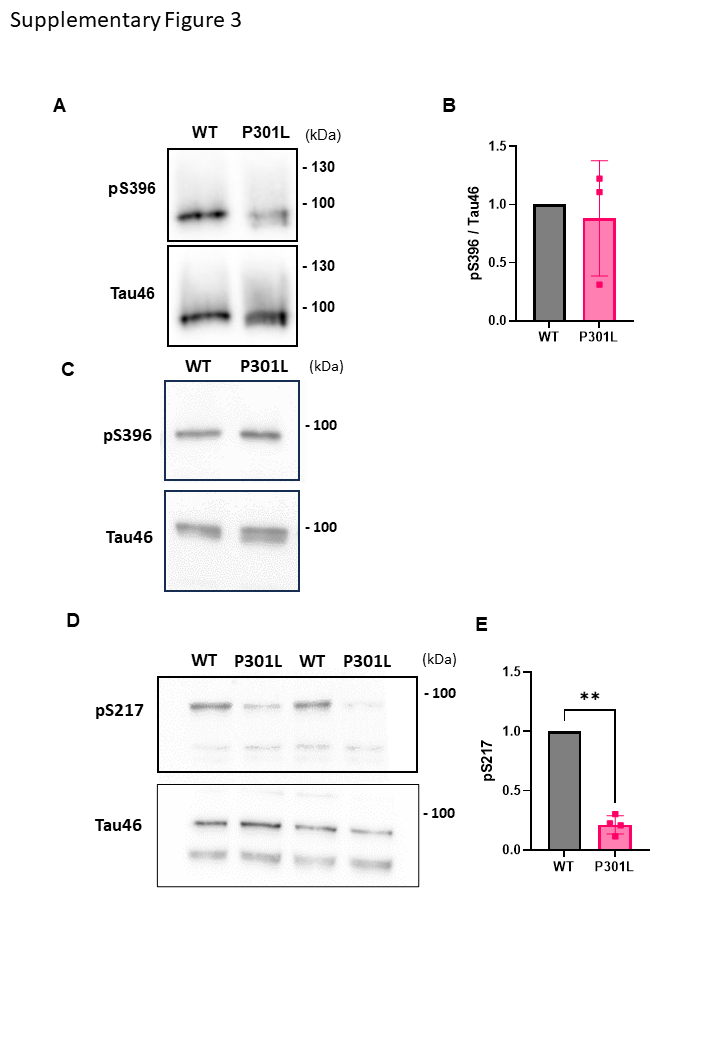


Supplementary Figure 3. Further exploration of differential tau phosphorylation. Immunoblotting of lysates from DIV13 neurons transduced with AAV-hSyn1-eGFP-TauWT or AAV-hSyn1-eGFP-TauP301L. A) Immunoblot probed with antibodies against tau phosphorylated at Ser396 or total tau (Tau46). B) Semi-quantitative analysis of pS396 levels normalised to Tau46 shows no significant difference between wild-type or mutant tau (ratio-paired t-test, P = 0.5817, n = 3). C) Additional immunoblot as in (A) highlighting variability in p396 between TauWT and TauP301L. D) Immunoblots probed with antibodies against tau phosphorylated at Ser217 (pS217) or total tau (Tau46) showing a notable decrease in mutant tau phosphorylation relative to wild-type with no notable difference in total tau levels. E) Semi-quantitative analysis of pS217 levels shows a significant decrease in phosphorylation at Ser217 (ratio-paired t-test, **p< 0.01, n = 4).


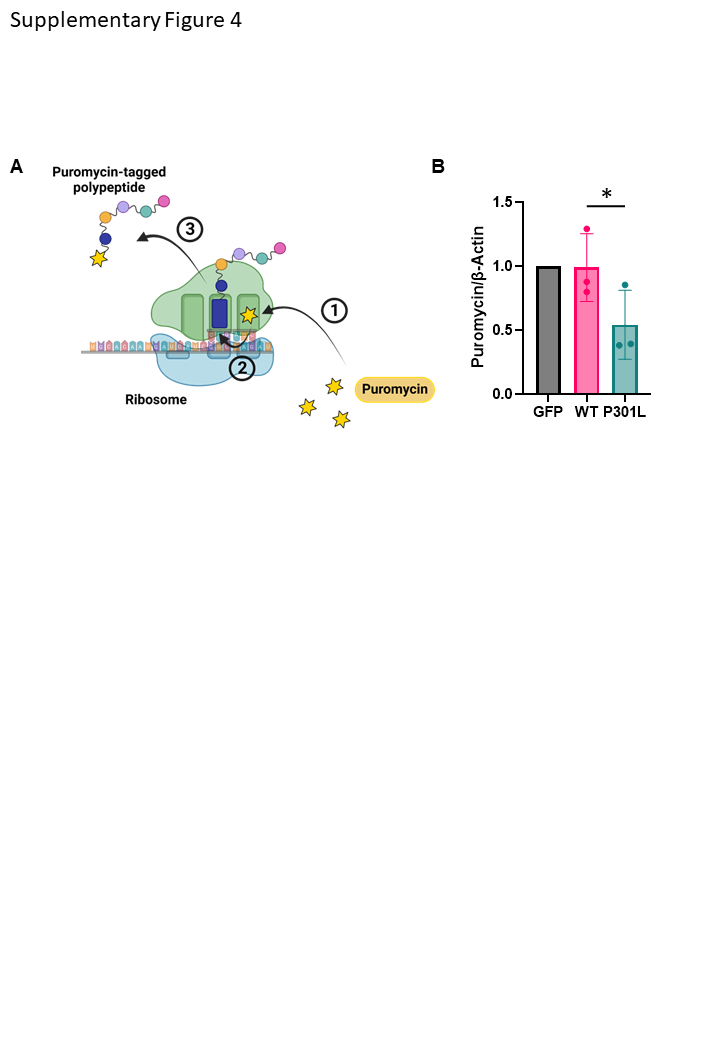


Supplementary Figure 4. eGFP-TauP301L overexpression may disrupt general translation. A) Puromycin is a tyrosyl-tRNA mimetic which can bind to the ribosomal A-site (1). Puromycin can then bind to peptidyl chains (2) which causes dissociation of the puromycin-tagged polypeptide from the ribosomal complex (3). The level of newly synthesized proteins can then be detected using antibodies against puromycin. Created with BioRender.com. B) Puromycin levels significantly decreased with eGFP-TauP301L relative to eGFP-TauWT but not eGFP (ratio-paired t-test, *p < 0.05, n =3).


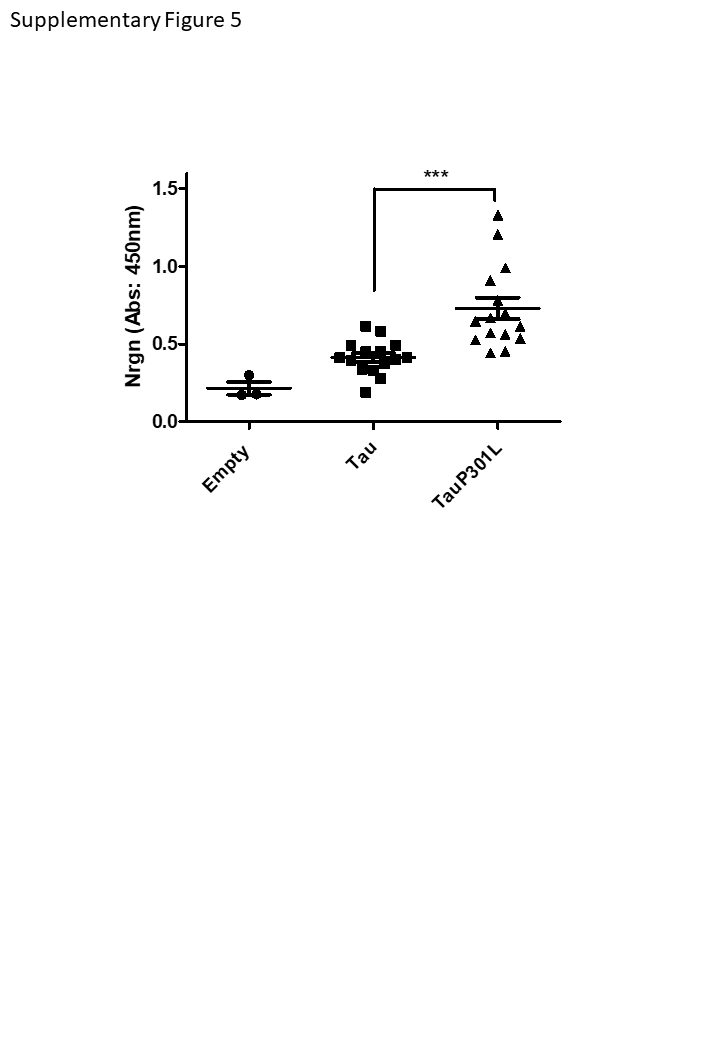


Supplementary Fig 5. eGFP-TauP301L overexpression increases levels of extracellular neurogranin. Neurogranin ELISA on media samples from eGFP (Empty, black circles, n=3), eGFP-TauWT (Tau, black squares, n=15) and eGFP-TauP301L (TauP301L, black triangles, n=15) transduced neurons. Neurogranin levels significantly increased in media of TauP301L expressed neurons compared to media of TauWT expressed neurons (One way ANOVA with Bonferroni’s Multiple Comparisons Test, ***p<0.001, n = 15 independent transductions). Horizontal bar indicates median.


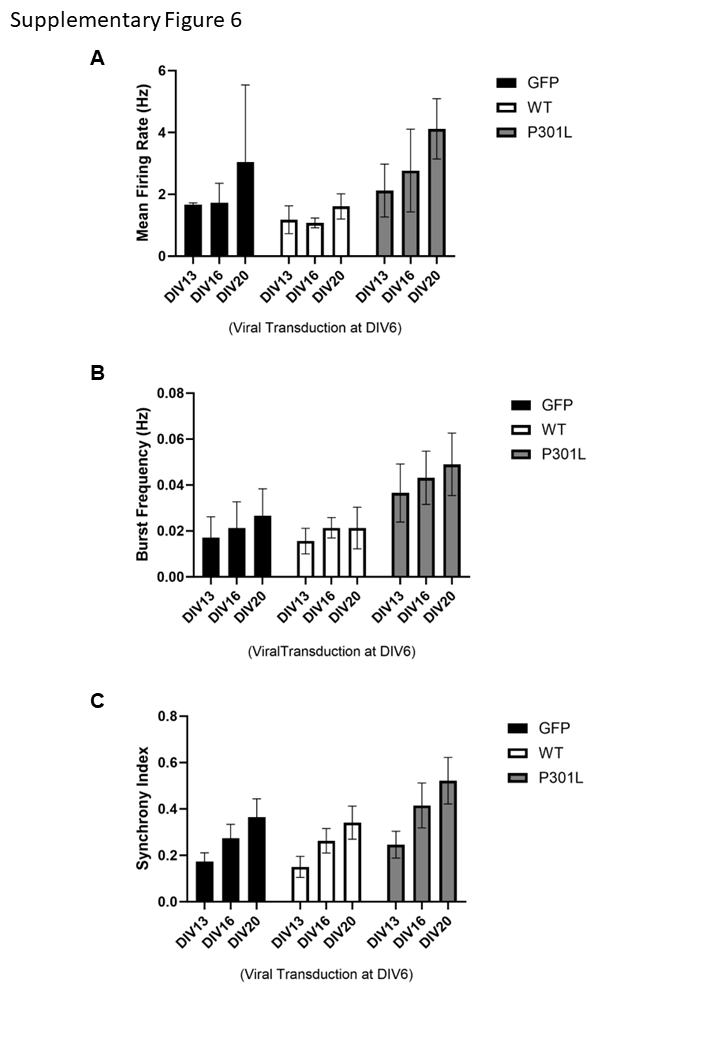


Supplementary Fig 6. Profile of neuronal network development in neurons transduced with viral vectors. Neurons were cultured in CytoView micro-electrode array (MEA) multiwell plates and transduced at DIV6 with AAV-hSyn1-eGFP (GFP; black bars) AAV-hSyn1-eGFP-TauWT (TauWT white bars) or AAV-hSyn1-eGFP-TauP301L (TauP301L, grey bars). Neurons were maintained for up to 20 days in vitro (DIV) and monitored for the appearance of spontaneous network activity at DIV13, DIV16 and DIV20 using the Maestro-Pro MEA system (Axion Biosystems). Recordings were made for 2 min from 5 independent wells of cells at each time-point shown. Data is presented as A) Mean Firing rate B) Burst Frequency C) Synchrony Index. Data is mean +/- SD n = 5 technical repeats.
