# Supplementary material for: TauP301L disengages from the proteosome core complex and neurogranin coincident with enhanced neuronal network excitability
Source: Cell Death Dis. 2024 Jun 18;15(6):429. doi: 10.1038/s41419-024-06815-2 (PMC11189525; doi:10.1038/s41419-024-06815-2)
Supplement: Supplementary file 2 — Proteomics summary list of differentially associated interactors [file 41419_2024_6815_MOESM2_ESM.pdf]

Supplementary Table 1

Information		Stats				Quantification							
Accession Information		P301L/WT				Log2 Normalised Abundances						P301L/WT	
BestAccession	Primary Gene Names	Replicate Number	LogFC	T-Test	FDR	GFP A	WT A	P301L A	GFP B	WT B	P301L B	Sample	
						126	127N	128N	129N	130N	131	A	B
Q9Z1G4	Atp6v0a1	2 v 2	0.98	4.76E-01	9.97E-01	3.41	6.16	6.24	3.20	4.29	6.18	1.01	1.44
P47962	Rpl5	2 v 2	0.90	4.34E-01	9.97E-01	2.54	6.60	6.77	2.46	5.19	6.82	1.03	1.31
P62301	Rps13	2 v 2	0.84	2.37E-01	9.97E-01	4.06	7.71	8.22	4.82	6.86	8.03	1.07	1.17
O09167	Rpl21	2 v 2	0.79	4.11E-01	9.97E-01	5.02	8.18	8.37	5.00	7.62	9.00	1.02	1.18
P12970	Rpl7a	2 v 2	0.77	3.10E-01	9.97E-01	6.07	9.95	10.31	5.90	9.23	10.40	1.04	1.13
P63017	Hspa8	2 v 2	0.71	1.56E-01	9.97E-01	9.18	13.70	14.23	8.77	13.71	14.59	1.04	1.06
P62855	Rps26	2 v 2	0.65	4.25E-01	9.97E-01	4.62	8.18	8.31	4.39	7.40	8.55	1.02	1.16
P35980	Rpl18	2 v 2	0.64	3.73E-01	9.97E-01	5.83	9.48	9.69	5.00	8.47	9.54	1.02	1.13
P62242	Rps8	2 v 2	0.63	3.29E-01	9.97E-01	4.76	8.31	8.58	4.50	7.40	8.39	1.03	1.13
P62911	Rpl32	2 v 2	0.61	4.56E-01	9.97E-01	5.68	8.92	9.00	4.80	7.82	8.96	1.01	1.15
Q80TA9	Epg5	2 v 2	0.57	3.89E-01	9.97E-01	1.00	5.87	6.04	0.77	5.93	6.89	1.03	1.16
A7MCT6	Etnk2	2 v 2	0.51	3.15E-01	9.97E-01	4.33	7.54	7.77	4.15	6.86	7.65	1.03	1.11
P62849	Rps24	2 v 2	0.49	6.81E-02	9.97E-01	5.87	8.81	9.35	6.07	8.83	9.27	1.06	1.05
Q8K284	Gtf3c1	2 v 2	0.48	2.73E-01	9.97E-01	1.72	7.35	7.61	2.41	7.03	7.73	1.04	1.10
O88809	Dcx	2 v 2	0.48	2.43E-01	9.97E-01	4.24	7.23	7.51	3.29	6.81	7.48	1.04	1.10
P41105	Rpl28	2 v 2	0.48	4.01E-01	9.97E-01	6.26	9.49	9.62	5.33	8.80	9.62	1.01	1.09
Q99LE1	Rilpl2	2 v 2	0.47	4.41E-01	9.97E-01	NA	7.70	8.56	1.14	8.77	8.85	1.11	1.01
P53026	Rpl10a	2 v 2	0.46	4.94E-01	9.97E-01	6.17	9.13	9.14	4.85	7.62	8.53	1.00	1.12
P99024	Tubb5	2 v 2	0.46	4.89E-01	9.97E-01	7.13	10.54	10.55	6.71	9.75	10.65	1.00	1.09
P62960	Ybx1	2 v 2	0.45	3.98E-01	9.97E-01	6.57	9.86	9.98	6.05	9.10	9.86	1.01	1.08
Q9D1R9	Rpl34	2 v 2	0.44	2.46E-01	9.97E-01	4.05	7.93	8.20	2.98	7.46	8.08	1.03	1.08
Q9D8E6	Rpl4	2 v 2	0.43	4.32E-01	9.97E-01	5.94	9.54	9.63	5.69	8.66	9.44	1.01	1.09
Q99PL5	Rrbp1	2 v 2	0.43	4.43E-01	9.97E-01	5.45	8.87	8.94	5.10	8.39	9.18	1.01	1.09
P84091	Ap2m1	2 v 2	0.41	4.82E-01	9.97E-01	6.13	9.69	9.71	5.58	8.71	9.51	1.00	1.09
Q61699	Hsph1	2 v 2	0.41	2.15E-01	9.97E-01	6.62	10.72	10.99	6.47	10.64	11.19	1.02	1.05
P14869	Rplp0	2 v 2	0.41	4.73E-01	9.97E-01	4.57	7.71	7.74	4.04	7.01	7.79	1.00	1.11
Q8VE33	Gdap1l1	2 v 2	0.38	3.88E-01	9.97E-01	3.25	6.63	7.28	2.85	6.83	6.95	1.10	1.02
P11798	Camk2a	2 v 2	0.37	3.04E-01	9.97E-01	5.55	8.78	8.95	5.27	7.76	8.32	1.02	1.07
P84078	Arf1	2 v 2	0.36	3.80E-01	9.97E-01	7.65	11.02	11.14	7.48	10.61	11.21	1.01	1.06
Q922R8	Pdia6	2 v 2	0.36	2.80E-01	9.97E-01	3.04	6.15	6.34	2.81	5.28	5.80	1.03	1.10
P25444	Rps2	2 v 2	0.34	4.42E-01	9.97E-01	5.84	9.35	9.40	4.96	8.09	8.71	1.01	1.08
Q93092	Taldo1	2 v 2	0.34	1.25E-01	9.97E-01	6.21	9.09	9.36	5.21	8.47	8.87	1.03	1.05
E9Q557	Dsp	2 v 2	0.34	2.14E-01	9.97E-01	6.87	8.34	8.79	5.40	8.20	8.42	1.05	1.03
P19253	Rpl13a	2 v 2	0.33	3.75E-01	9.97E-01	5.81	9.08	9.19	5.54	8.28	8.84	1.01	1.07
Q91VR7	Map1lc3a	2 v 2	0.33	4.98E-01	9.97E-01	4.56	7.30	7.30	3.46	6.74	7.39	1.00	1.10
P68040	Rack1	2 v 2	0.32	6.28E-02	9.97E-01	3.47	6.01	6.36	2.93	6.04	6.33	1.06	1.05
P62827	Ran	2 v 2	0.32	3.49E-01	9.97E-01	6.09	9.34	9.46	5.99	8.70	9.21	1.01	1.06
P27659	Rpl3	2 v 2	0.31	4.29E-01	9.97E-01	5.65	9.66	9.72	5.48	8.73	9.29	1.01	1.06
Q9CZ30	Ola1	2 v 2	0.31	3.37E-01	9.97E-01	4.33	7.31	7.80	3.89	7.01	7.14	1.07	1.02
Q6ZWZ4	Rpl36	2 v 2	0.30	5.55E-02	9.97E-01	3.15	6.24	6.52	3.20	5.84	6.17	1.04	1.06
Q9CZX8	Rps19	2 v 2	0.30	4.96E-01	9.97E-01	6.16	8.92	8.92	5.32	8.27	8.87	1.00	1.07
Q9WV60	Gsk3b	2 v 2	0.30	4.47E-01	9.97E-01	3.82	7.66	7.71	3.80	6.98	7.53	1.01	1.08
Q9D6F9	Tubb4a	2 v 2	0.28	3.81E-01	9.97E-01	6.07	9.51	9.60	5.62	9.23	9.70	1.01	1.05
Q8VEM8	Slc25a3	2 v 2	0.28	3.45E-01	9.97E-01	6.41	9.37	9.48	6.33	8.19	8.64	1.01	1.05
Q61990	Pcbp2	2 v 2	0.28	4.22E-01	9.97E-01	4.54	7.34	7.41	3.98	6.76	7.25	1.01	1.07
Q9Z1A1	Tfg	2 v 2	0.28	1.62E-01	9.97E-01	3.96	6.73	6.93	3.55	6.52	6.86	1.03	1.05
P62751	Rpl23a	2 v 2	0.27	1.70E-01	9.97E-01	6.90	10.31	10.51	7.06	9.96	10.31	1.02	1.04
Q03265	Atp5f1a	2 v 2	0.26	4.47E-01	9.97E-01	8.45	11.70	11.74	8.28	11.32	11.81	1.00	1.04
P47911	Rpl6	2 v 2	0.26	4.03E-01	9.97E-01	6.75	10.28	10.35	7.72	10.37	10.83	1.01	1.04
Q8C0E2	Vps26b	2 v 2	0.26	4.70E-01	9.97E-01	2.68	6.31	6.34	1.38	5.45	5.95	1.00	1.09
Q9CWF2	Tubb2b	2 v 2	0.26	2.83E-01	9.97E-01	5.71	9.13	9.26	5.68	8.90	9.28	1.01	1.04
P62267	Rps23	2 v 2	0.25	4.73E-01	9.97E-01	5.42	8.70	8.72	6.76	9.42	9.90	1.00	1.05
P62702	Rps4x	2 v 2	0.24	4.59E-01	9.97E-01	5.94	9.53	9.56	5.87	8.72	9.17	1.00	1.05
Q9Z0E0	Ncdn	2 v 2	0.22	3.89E-01	9.97E-01	5.58	8.60	8.67	4.36	7.55	7.92	1.01	1.05
P62754	Rps6	2 v 2	0.21	3.24E-01	9.97E-01	6.00	9.20	9.30	6.51	9.06	9.39	1.01	1.04
Q9CPR4	Rpl17	2 v 2	0.21	4.94E-01	9.97E-01	4.50	8.22	8.23	4.71	7.55	7.97	1.00	1.05
P97351	Rps3a	2 v 2	0.21	2.97E-01	9.97E-01	7.75	11.04	11.14	7.87	10.61	10.91	1.01	1.03
P16546	Sptan1	2 v 2	0.20	8.28E-02	9.97E-01	6.39	9.20	9.37	6.09	9.22	9.45	1.02	1.03
Q9JJV2	Pfn2	2 v 2	0.20	1.01E-01	9.97E-01	6.56	9.77	10.00	6.09	9.06	9.23	1.02	1.02
P61358	Rpl27	2 v 2	0.20	2.01E-02	9.97E-01	4.39	7.74	7.94	4.82	7.59	7.78	1.03	1.03
P61358	Rpl27	2 v 2	0.20	2.01E-02	9.97E-01	4.39	7.74	7.94	4.82	7.59	7.78	1.03	1.03
P62717	Rpl18a	2 v 2	0.20	4.41E-01	9.97E-01	5.61	7.88	7.92	5.21	7.38	7.74	1.00	1.05
P68510	Ywhah	2 v 2	0.19	1.44E-02	9.97E-01	6.74	10.37	10.55	6.65	10.43	10.63	1.02	1.02
Q80XN0	Bdh1	2 v 2	0.18	2.63E-01	9.97E-01	3.62	7.18	7.28	3.74	6.82	7.08	1.01	1.04
P50580	Pa2g4	2 v 2	0.18	2.55E-01	9.97E-01	3.38	6.72	6.82	2.68	7.03	7.28	1.02	1.04
Q61316	Hspa4	2 v 2	0.17	3.25E-01	9.97E-01	6.04	9.20	9.48	5.86	9.07	9.15	1.03	1.01
Q9D883	U2af1	2 v 2	0.17	1.76E-01	9.97E-01	5.30	8.02	8.14	5.28	8.05	8.27	1.01	1.03
Q8R081	Hnrnpl	2 v 2	0.14	5.44E-02	9.97E-01	3.15	6.06	6.21	3.36	5.38	5.51	1.02	1.02
Q8K2C9	Hacd3	2 v 2	0.13	2.37E-01	9.97E-01	3.52	6.75	6.94	3.10	6.27	6.35	1.03	1.01
O88935	Syn1	2 v 2	0.13	3.13E-01	9.97E-01	3.22	6.62	6.68	3.43	6.09	6.29	1.01	1.03
Q11011	Npepps	2 v 2	0.13	4.00E-01	9.97E-01	4.81	8.40	8.43	4.81	8.25	8.47	1.00	1.03
P56376	Acyp1	2 v 2	0.13	3.64E-01	9.97E-01	3.79	6.92	7.13	4.08	6.88	6.93	1.03	1.01
Q9Z204	Hnrnpc	2 v 2	0.12	5.66E-02	9.97E-01	3.00	6.61	6.72	3.10	6.55	6.68	1.02	1.02
Q9CR57	Rpl14	2 v 2	0.11	7.99E-02	9.97E-01	5.49	7.93	8.03	5.32	7.51	7.64	1.01	1.02
P97300	Nptn	2 v 2	0.09	4.30E-01	9.97E-01	3.05	7.53	7.55	3.66	7.81	7.98	1.00	1.02
P47915	Rpl29	2 v 2	0.09	3.56E-01	9.97E-01	6.53	9.78	9.81	7.09	9.77	9.92	1.00	1.02
Q9CZ13	Uqcrc1	2 v 2	0.08	1.72E-01	9.97E-01	5.16	8.03	8.13	4.88	7.75	7.81	1.01	1.01
P27661	H2ax	2 v 2	0.06	2.64E-03	9.97E-01	4.19	7.77	7.83	4.22	7.03	7.09	1.01	1.01
Q501J6	Ddx17	2 v 2	0.05	4.53E-01	9.97E-01	2.96	6.02	6.02	3.31	6.21	6.30	1.00	1.01
Q8R016	Blmh	2 v 2	0.05	3.70E-01	9.97E-01	5.35	7.57	7.65	4.41	7.39	7.41	1.01	1.00
Q9DC70	Ndufs7	2 v 2	0.05	3.84E-01	9.97E-01	3.47	6.76	6.84	3.95	6.81	6.82	1.01	1.00

Information		Stats				Quantification							
Accession Information		P301L/WT				Log2 Normalised Abundances						P301L/WT	
BestAccession	Primary Gene Names	Replicate Number	LogFC	T-Test	FDR	GFP A	WT A	P301L A	GFP B	WT B	P301L B	Sample	
						126	127N	128N	129N	130N	131	A	B
P60761	Nrgn	2 v 2	-2.06	1.61E-01	9.97E-01	2.98	8.19	5.59	1.00	5.47	3.94	0.68	0.72
P35821	Ptpn1	2 v 2	-1.15	3.35E-01	9.97E-01	5.40	9.82	9.34	7.60	9.44	7.62	0.95	0.81
Q8R1I1	Uqcr10	2 v 2	-1.14	1.87E-01	9.97E-01	3.43	7.00	6.21	3.75	7.19	5.71	0.89	0.79
Q9D0K2	Oxct1	2 v 2	-1.09	1.91E-01	9.97E-01	5.17	8.79	8.04	4.52	8.95	7.53	0.91	0.84
Q9D3D9	Atp5f1d	2 v 2	-0.96	1.53E-01	9.97E-01	4.06	6.76	5.56	4.60	6.62	5.89	0.82	0.89
Q9R1P1	Psmb3	2 v 2	-0.75	2.73E-01	9.97E-01	3.28	6.77	6.36	2.89	6.25	5.16	0.94	0.83
Q569Z6	Thrap3	2 v 2	-0.72	3.20E-01	9.97E-01	2.98	6.58	6.26	4.06	7.21	6.10	0.95	0.85
Q62446	Fkbp3	2 v 2	-0.69	3.70E-02	9.97E-01	3.14	6.62	5.97	1.54	6.61	5.87	0.90	0.89
Q9CQW1	Ykt6	2 v 2	-0.69	9.80E-02	9.97E-01	5.04	7.99	7.19	5.24	8.09	7.51	0.90	0.93
P61922	Abat	2 v 2	-0.66	1.78E-01	9.97E-01	2.93	6.01	5.16	1.81	6.61	6.14	0.86	0.93
O55234	Psmb5	2 v 2	-0.66	1.94E-01	9.97E-01	4.04	6.98	6.53	4.14	7.02	6.15	0.94	0.88
Q8BX17	Gemin5	2 v 2	-0.65	2.36E-01	9.97E-01	5.51	8.27	7.87	5.62	8.79	7.89	0.95	0.90
P62264	Rps14	2 v 2	-0.57	1.06E-01	9.97E-01	6.17	9.57	8.91	6.12	9.41	8.94	0.93	0.95
P01869	Ighg1	2 v 2	-0.54	1.51E-01	9.97E-01	3.90	6.61	6.20	3.78	6.11	5.43	0.94	0.89
P62774	Mtpn	2 v 2	-0.54	1.73E-01	9.97E-01	4.70	8.07	7.38	4.33	7.47	7.08	0.91	0.95
P15105	Glul	2 v 2	-0.48	2.88E-01	9.97E-01	7.14	10.30	10.05	6.92	9.86	9.15	0.98	0.93
P63328	Ppp3ca	2 v 2	-0.47	2.79E-02	9.97E-01	4.91	8.31	7.81	4.47	7.59	7.14	0.94	0.94
Q8BHL3	Tbc1d10b	2 v 2	-0.46	2.68E-01	9.97E-01	5.21	6.87	6.62	4.76	7.44	6.77	0.96	0.91
P70699	Gaa	2 v 2	-0.46	3.78E-01	9.97E-01	4.66	7.57	7.42	4.94	8.07	7.30	0.98	0.90
P62984	Uba52	2 v 2	-0.46	3.81E-01	9.97E-01	5.21	8.13	7.36	6.41	9.07	8.92	0.90	0.98
P10649	Gstm1	2 v 2	-0.46	1.74E-01	9.97E-01	5.84	8.82	8.49	5.58	8.42	7.84	0.96	0.93
P70168	Kpnb1	2 v 2	-0.43	1.36E-01	9.97E-01	2.68	6.80	6.28	2.61	6.81	6.48	0.92	0.95
P12025	Mdk	2 v 2	-0.42	2.51E-01	9.97E-01	7.79	10.59	10.35	8.16	10.80	10.21	0.98	0.95
Q3UHX2	Pdap1	2 v 2	-0.41	4.16E-01	9.97E-01	4.00	7.24	6.52	4.22	6.96	6.86	0.90	0.99
Q9R111	Gda	2 v 2	-0.40	9.17E-02	9.97E-01	3.99	7.70	7.24	4.69	7.91	7.56	0.94	0.96
Q99ME9	Gtpbp4	2 v 2	-0.40	2.67E-01	9.97E-01	5.63	8.53	8.31	7.06	9.64	9.05	0.97	0.94
Q64433	Hspe1	2 v 2	-0.39	1.84E-01	9.97E-01	6.31	9.43	8.93	6.15	8.96	8.69	0.95	0.97
Q9QUM9	Psma6	2 v 2	-0.37	3.29E-01	9.97E-01	4.15	7.40	6.83	3.34	6.22	6.06	0.92	0.97
P35979	Rpl12	2 v 2	-0.36	2.23E-01	9.97E-01	4.34	7.57	7.07	4.34	7.28	7.05	0.93	0.97
Q9CQI6	Cotl1	2 v 2	-0.36	9.32E-02	9.97E-01	3.83	7.05	6.63	2.87	6.05	5.74	0.94	0.95
Q8K0T0	Rtn1	2 v 2	-0.36	7.27E-02	9.97E-01	5.31	8.41	8.01	4.67	8.10	7.78	0.95	0.96
Q6PHN9	Rab35	2 v 2	-0.36	9.78E-02	9.97E-01	3.72	6.97	6.56	3.39	6.87	6.57	0.94	0.96
P62862	Fau	2 v 2	-0.34	9.44E-02	9.97E-01	7.75	10.39	10.10	8.09	10.60	10.20	0.97	0.96
P54227	Stmn1	2 v 2	-0.33	4.86E-01	9.97E-01	8.28	11.38	10.73	7.54	10.65	10.64	0.94	1.00
Q9QYR6	Map1a	2 v 2	-0.33	1.30E-01	9.97E-01	5.67	9.61	9.21	6.94	9.26	8.99	0.96	0.97
P49722	Psma2	2 v 2	-0.33	3.68E-01	9.97E-01	4.04	7.00	6.46	3.81	5.79	5.68	0.92	0.98
O35143	Atp5if1	2 v 2	-0.33	1.43E-01	9.97E-01	5.78	8.71	8.31	5.15	8.29	8.04	0.95	0.97
P35803	Gpm6b	2 v 2	-0.32	2.78E-01	9.97E-01	4.42	7.60	7.12	4.73	7.86	7.69	0.94	0.98
Q9Z2Q6	Septin5	2 v 2	-0.32	2.94E-01	9.97E-01	4.06	7.46	6.97	4.19	7.23	7.07	0.94	0.98
E9PZF0	Gm20390	2 v 2	-0.32	3.94E-01	9.97E-01	7.06	10.20	9.65	6.59	9.52	9.43	0.95	0.99
Q9CY58	Serbp1	2 v 2	-0.32	3.23E-01	9.97E-01	6.32	9.45	8.95	6.96	9.13	8.99	0.95	0.98
Q4VAA2	Cdv3	2 v 2	-0.31	3.05E-01	9.97E-01	4.50	7.56	7.09	4.39	7.18	7.03	0.94	0.98
Q61937	Npm1	2 v 2	-0.31	4.39E-01	9.97E-01	5.61	8.68	8.12	5.55	8.16	8.10	0.94	0.99
Q64511	Top2b	2 v 2	-0.31	3.44E-02	9.97E-01	5.31	8.43	8.14	5.98	8.99	8.67	0.97	0.96
Q9JKD3	Scamp5	2 v 2	-0.31	2.39E-01	9.97E-01	4.17	7.44	7.26	5.05	7.87	7.44	0.98	0.95
P17095	Hmga1	2 v 2	-0.30	2.80E-01	9.97E-01	6.54	9.54	9.11	6.60	9.48	9.33	0.95	0.98
P61027	Rab10	2 v 2	-0.29	8.30E-02	9.97E-01	5.13	8.30	7.97	4.47	7.51	7.26	0.96	0.97
Q91V61	Sfxn3	2 v 2	-0.29	3.00E-01	9.97E-01	4.78	7.62	7.48	4.87	7.78	7.35	0.98	0.94
G5E829	Atp2b1	2 v 2	-0.29	2.07E-01	9.97E-01	3.07	6.09	5.70	3.47	6.39	6.20	0.94	0.97
P08228	Sod1	2 v 2	-0.28	3.61E-01	9.97E-01	5.68	8.61	8.15	5.12	7.82	7.71	0.95	0.99
P63089	Ptn	2 v 2	-0.28	6.62E-02	9.97E-01	8.75	11.32	11.07	8.77	11.31	11.00	0.98	0.97
Q62277	Syp	2 v 2	-0.27	3.79E-01	9.97E-01	5.03	8.63	8.18	5.73	9.04	8.95	0.95	0.99
Q7TQI3	Otub1	2 v 2	-0.27	5.36E-02	9.97E-01	4.86	8.26	7.97	4.73	7.88	7.63	0.96	0.97
Q62426	Cstb	2 v 2	-0.24	2.19E-01	9.97E-01	4.84	8.09	7.76	3.04	7.17	7.02	0.96	0.98
Q8BKT2	Hes7	2 v 2	-0.24	2.53E-01	9.97E-01	4.94	7.91	7.77	4.68	7.46	7.11	0.98	0.95
Q9Z2I9	Sucla2	2 v 2	-0.22	4.89E-01	9.97E-01	3.14	6.57	6.14	2.83	5.26	5.25	0.93	1.00
Q61687	Atrx	2 v 2	-0.22	3.92E-01	9.97E-01	3.05	5.95	5.88	4.57	6.79	6.41	0.99	0.94
P80313	Cct7	2 v 2	-0.22	9.12E-02	9.97E-01	5.54	8.85	8.60	5.21	8.41	8.22	0.97	0.98
Q9DCT2	Ndufs3	2 v 2	-0.21	2.90E-01	9.97E-01	4.57	8.17	7.86	4.57	8.02	7.91	0.96	0.99
Q61792	Lasp1	2 v 2	-0.20	4.40E-01	9.97E-01	5.89	8.89	8.51	5.50	8.39	8.36	0.96	1.00
P09405	Ncl	2 v 2	-0.20	1.41E-01	9.97E-01	3.66	7.00	6.76	3.63	6.77	6.61	0.97	0.98
O88447	Klc1	2 v 2	-0.19	4.61E-01	9.97E-01	5.53	8.43	8.41	5.42	8.62	8.27	1.00	0.96
P30681	Hmgb2	2 v 2	-0.18	2.84E-01	9.97E-01	5.44	8.82	8.55	4.87	7.84	7.74	0.97	0.99
F8VQC1	Srp72	2 v 2	-0.18	1.07E-01	9.97E-01	6.24	9.13	8.98	6.35	9.29	9.08	0.98	0.98
Q3UL36	Arglu1	2 v 2	-0.18	4.19E-02	9.97E-01	5.63	8.47	8.30	5.98	8.64	8.45	0.98	0.98
O35643	Ap1b1	2 v 2	-0.17	5.06E-02	9.97E-01	3.20	6.93	6.77	2.81	6.53	6.34	0.98	0.97
P83882	Rpl36a	2 v 2	-0.17	1.47E-02	9.97E-01	3.20	6.31	6.14	3.98	6.62	6.45	0.97	0.98
Q8VDN2	Atp1a1	2 v 2	-0.16	2.94E-01	9.97E-01	4.14	7.29	7.20	4.47	7.56	7.31	0.99	0.97
P62274	Rps29	2 v 2	-0.16	4.10E-01	9.97E-01	4.43	7.22	6.94	4.62	6.65	6.61	0.96	0.99
Q9Z2U0	Psma7	2 v 2	-0.16	2.59E-01	9.97E-01	5.53	8.49	8.26	5.85	7.63	7.54	0.97	0.99
Q9Z315	Sart1	2 v 2	-0.16	3.96E-02	9.97E-01	4.95	7.98	7.82	5.19	7.96	7.82	0.98	0.98
P40124	Cap1	2 v 2	-0.15	4.82E-01	9.97E-01	6.09	9.41	9.12	5.54	8.64	8.63	0.97	1.00
O88892	Serf1	2 v 2	-0.14	3.58E-01	9.97E-01	3.86	6.96	6.90	4.24	7.34	7.11	0.99	0.97
P51880	Fabp7	2 v 2	-0.14	4.32E-01	9.97E-01	6.13	9.46	9.22	6.36	9.33	9.30	0.97	1.00
P61079	Ube2d3	2 v 2	-0.13	5.95E-02	9.97E-01	4.23	7.33	7.18	3.64	6.35	6.23	0.98	0.98
Q8BFR5	Tufm	2 v 2	-0.13	4.96E-01	9.97E-01	5.42	8.98	8.71	5.45	8.39	8.38	0.97	1.00
Q91ZZ3	Sncb	2 v 2	-0.13	4.27E-01	9.97E-01	3.10	6.73	6.50	3.77	6.71	6.68	0.97	1.00
Q9DCT8	Crip2	2 v 2	-0.12	4.95E-01	9.97E-01	4.74	7.85	7.85	5.03	7.90	7.66	1.00	0.97
P11031	Sub1	2 v 2	-0.12	3.18E-01	9.97E-01	5.12	7.79	7.61	4.34	7.55	7.49	0.98	0.99
Q5SUF2	Luc7l3	2 v 2	-0.12	3.86E-01	9.97E-01	4.85	7.76	7.56	4.79	8.01	7.97	0.97	1.00
Q6ZWV7	Rpl35	2 v 2	-0.11	3.50E-01	9.97E-01	7.12	9.97	9.93	7.52	10.18	10.00	1.00	0.98
P63325	Rps10	2 v 2	-0.10	4.64E-01	9.97E-01	5.50	8.70	8.51	5.89	8.58	8.57	0.98	1.00
P16125	Ldhb	2 v 2	-0.10	2.30E-01	9.97E-01	6.40	9.49	9.43	5.63	8.98	8.85	0.99	0.99
P62852	Rps25	2 v 2	-0.07	4.79E-01	9.97E-01	8.04	10.66	10.53	8.59	11.24	11.23	0.99	1.00
F6R7E8	Gm2663	2 v 2	-0.01	4.02E-01	9.97E-01	3.17	6.04	6.03	4.20	6.40	6.40	1.00	1.00
