# Supplementary figures and images for: TauP301L disengages from the proteosome core complex and neurogranin coincident with enhanced neuronal network excitability
Source: Cell Death Dis. 2024 Jun 18;15(6):429. doi: 10.1038/s41419-024-06815-2 (PMC11189525; doi:10.1038/s41419-024-06815-2)

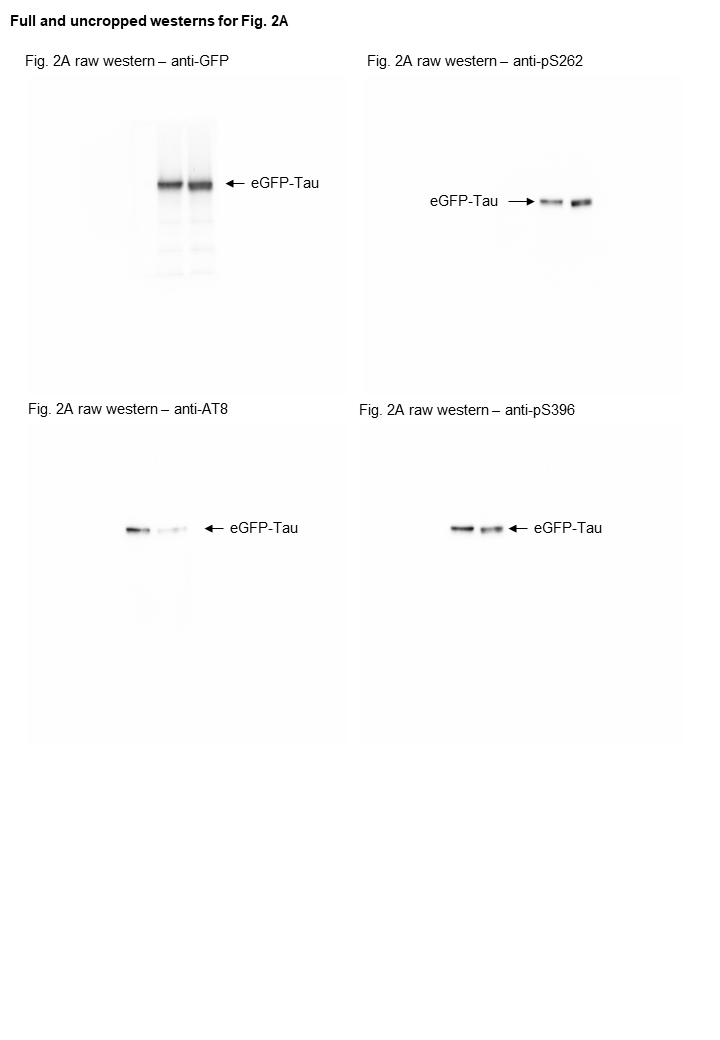


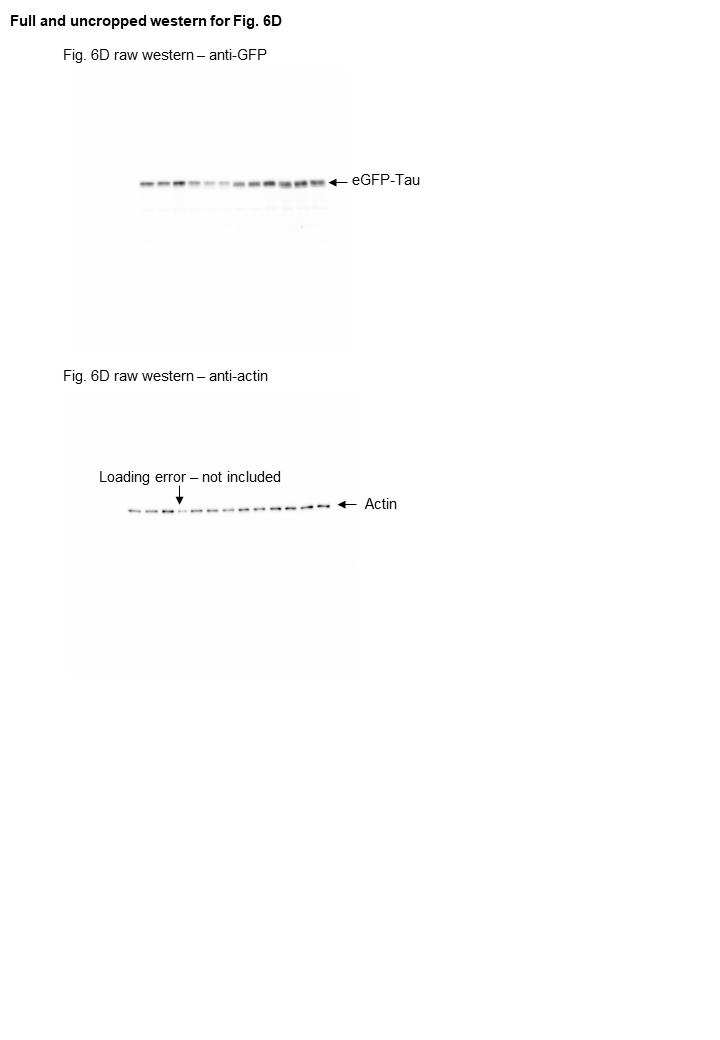


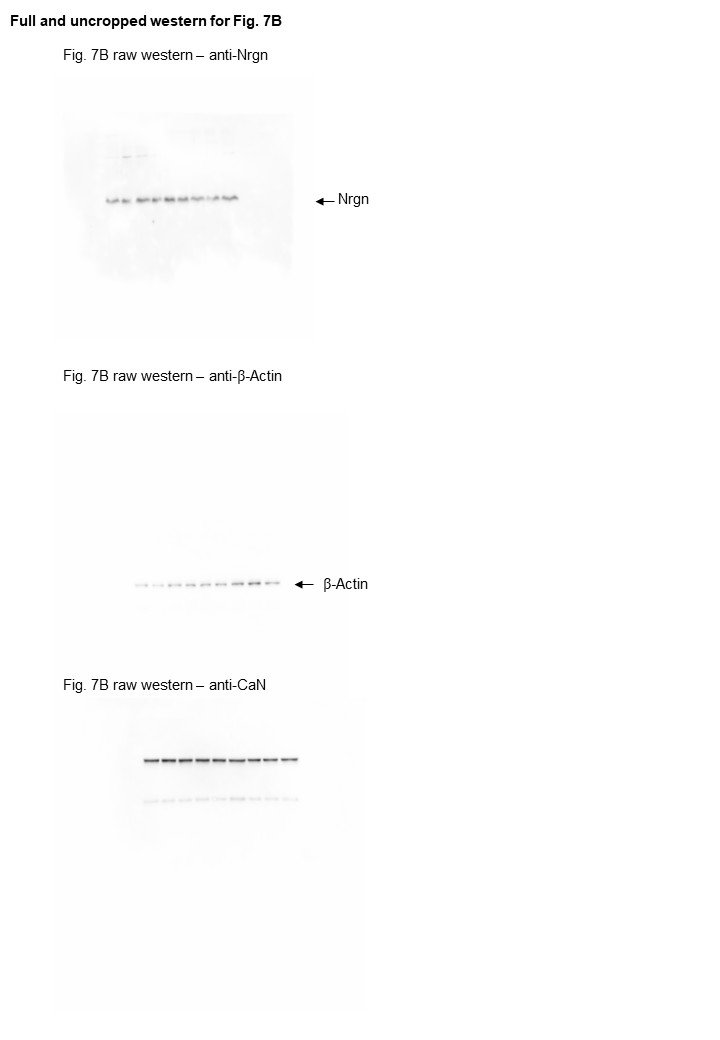

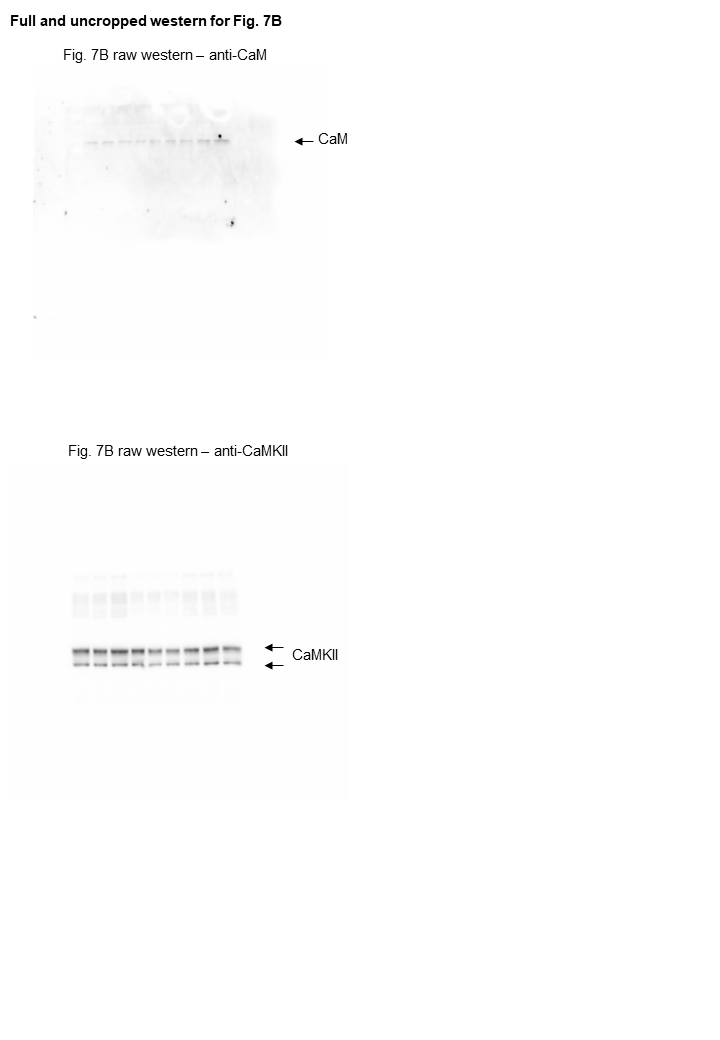

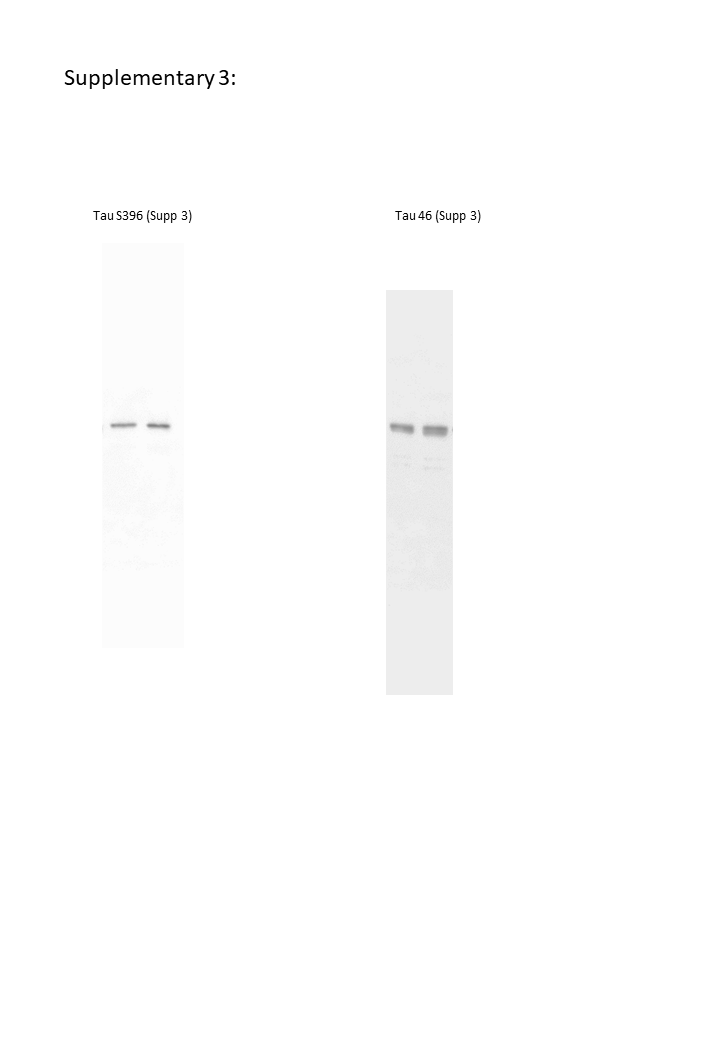

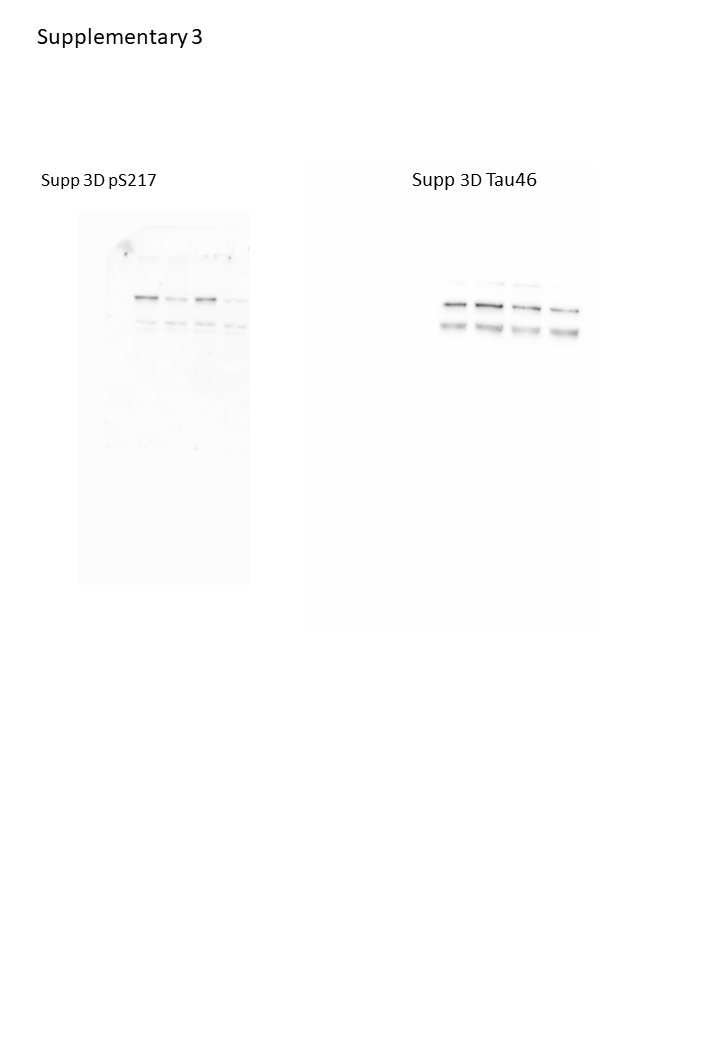

Supplement: Supplementary file 4 — Uncropped Western blots [file 41419_2024_6815_MOESM4_ESM.docx]
